# Artificial intelligence‐driven anticancer peptide discovery

**DOI:** 10.1002/imo2.70063

**Published:** 2025-11-05

**Authors:** Junrui Wu, Shuaiqi Ji, Kashif Iqbal Sahibzada, Mengxue Lou, Feiyu An, Wenqian Li, Jiawei Guo, Taowei Zhang, Xinyi Zhang, Yilin Chou, Henan Zhang, Hao Jin, Teng Ma, Weichi Liu, Begali Alikulov, Natalia Alekseevna Golovneva, Hooi Ling Foo, Issayeva Kuralay, Zhihong Sun, Dongqing Wei, Rina Wu

**Affiliations:** ^1^ College of Food Science Shenyang Agricultural University, National Agricultural Environmental Microbial Germplasm Resource Bank, Liaoning Engineering Research Center of Food Fermentation Technology, Shenyang Key Laboratory of Microbial Fermentation Technology Innovation Shenyang PR China; ^2^ College of Biological Engineering Henan University of Technology Zhengzhou PR China; ^3^ Department of Health Professional Technologies, Faculty of Allied Health Sciences The University of Lahore Lahore Pakistan; ^4^ State Key Laboratory of Microbial Metabolism, Joint International Research Laboratory of Metabolic and Developmental Sciences, School of Life Sciences and Biotechnology Shanghai Jiao Tong University Shanghai PR China; ^5^ Key Laboratory of Dairy Biotechnology and Engineering, Ministry of Education Inner Mongolia Agricultural University Hohhot PR China; ^6^ Department of Biotechnology Samarkand State University Samarkand Uzbekistan; ^7^ Institute of Microbiology of the National Academy of Sciences of Belarus Minsk Belarus; ^8^ Department of Bioprocess Technology, Faculty of Biotechnology and Biomolecular Sciences Universiti Putra Malaysia Serdang Selangor Malaysia; ^9^ Lactic Acid Bacteria Biota Technology Research Program, Research Laboratory of Probiotics and Cancer Therapeutics, UPM‐MAKNA Cancer Research Laboratory (CANRES), Institute of Bioscience Universiti Putra Malaysia Serdang Selangor Malaysia; ^10^ Department of Biotechnology Toraighyrov University Pavlodar Kazakhstan

**Keywords:** anticancer peptides, artificial intelligence, cancer, machine learning

## Abstract

Cancer has become a major global health threat. Despite advances in modern medicine, current therapeutic strategies still face many limitations. Anticancer peptides (ACPs), due to their high selectivity, low toxicity, and multitarget effects, have gradually become a research focus in the development of novel peptide‐based anticancer drugs. However, traditional screening methods are constrained by their low efficiency, high costs, and technical complexity, limiting their capacity to meet the demands of high‐throughput applications. Artificial intelligence (AI) has provided new methods to address these challenges, significantly improving the efficiency and accuracy of ACP screening through the application of machine learning and deep learning algorithms. To further enhance the application of AI in ACP screening, the advantages and limitations of 68 AI models used for ACP screening are systematically summarized. AI models show considerable potential for discovering ACPs, but most of these models lack interpretability and wet‐laboratory validation, which hinder the credibility and practical effectiveness of AI‐based ACP screening. Therefore, we presented a comprehensive ACP screening framework based on AI models. The presented framework includes data collection and organization, feature extraction, model construction, model interpretability analysis, and experimental validation. Additionally, we integrated this screening framework with multi‐omics and other biotechnologies to promote the translation of AI‐selected ACPs to the clinic. The presented AI‐based ACP screening framework can accelerate the ACP development, increase ACP screening efficiency, and promote clinical ACP application.

## Highlights

- Summarized 68 anticancer peptide (ACP) models.
- Artificial Intelligence (AI) interpretability and wet‐laboratory validation enhance ACP mining trustworthiness.
- AI combined with multi‐omics and biotechnologies drives ACP clinical translation.
- Summarized a comprehensive AI‐driven ACP screening framework.

## Graphical abstract

This review systematically summarized 68 artificial intelligence (AI) models for anticancer peptide (ACP) screening and presented a comprehensive AI‐based framework. The framework consists of six components: data collection and organization, feature extraction, model construction, interpretability analysis, experimental validation, and integration with multi‐omics and biotechnologies. This strategy improves screening efficiency and accuracy, addresses the limitations of interpretability and wet‐lab validation, and accelerates the translation of AI‐discovered ACPs into clinical applications.

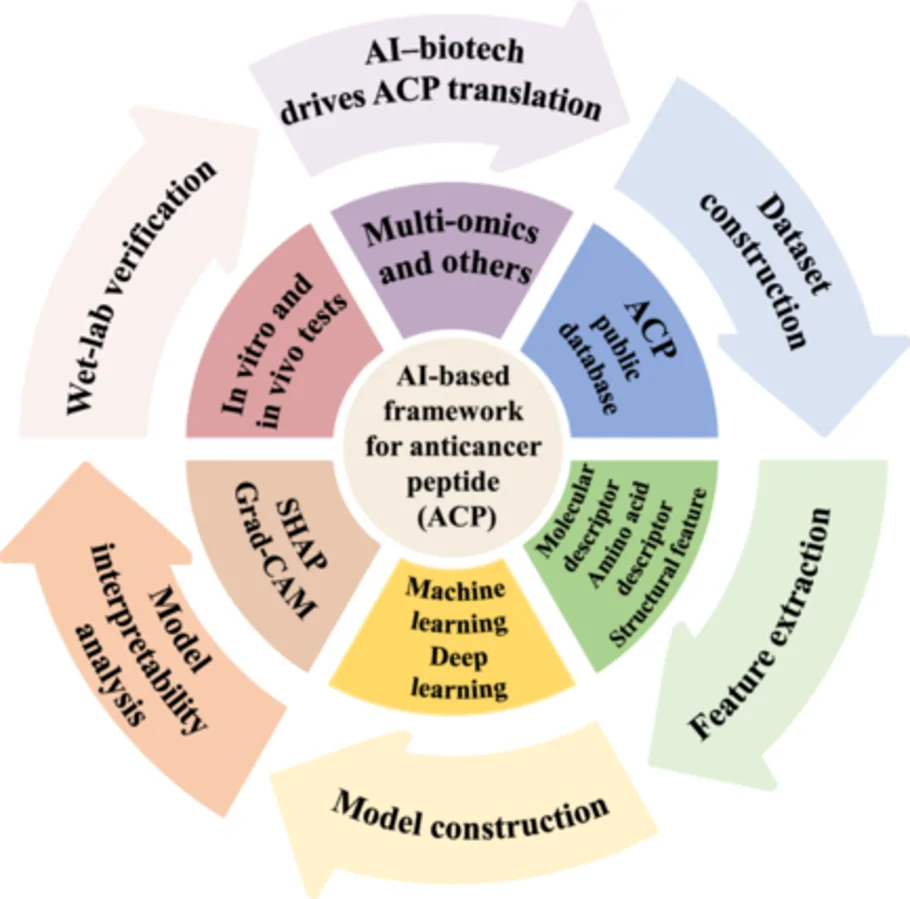

## 1 INTRODUCTION

Cancer is one of the leading causes of death worldwide, with its high incidence and mortality rates posing a significant burden on global public health systems. According to the International Agency for Research on Cancer, there were 20 million new cancer cases globally in 2022, which is expected to exceed 35 million by the middle of this century [1]. Despite significant advances in cancer diagnosis and treatment, such as the application of targeted therapies and immunotherapies, current therapeutic approaches still face many limitations. Conventional chemotherapy, currently the most widely used treatment method, achieves its anticancer effect by inhibiting tumor cell proliferation. But chemotherapeutic drugs often lack cell specificity, due to which, they damage not only cancer cells but also normal tissue cells, leading to severe side effects, such as bone marrow suppression, gastrointestinal toxicity, and immune dysfunction, causing significant harm to human health [2]. Additionally, during treatment, cancer cells may develop resistance to drugs through mechanisms such as gene mutations, activation of drug efflux pumps, or disappearance or mutation of therapeutic targets, resulting in reduced treatment efficacy or even complete treatment failure [3]. This dual dilemma of drug resistance and toxicity severely limits the effectiveness of cancer drugs. Therefore, developing novel anticancer drugs with higher selectivity and lower toxicity to overcome existing treatment bottlenecks has become a key focus of cancer research.

Anticancer peptides (ACPs) are a class of bioactive peptides characterized by their anticancer activity [4]. Compared to conventional chemotherapeutic drugs, ACPs exhibit high selectivity and low toxicity. They can specifically recognize receptors or membrane components on the surface of cancer cells, thereby achieving the precise targeting of cancer cells while reducing harm to normal tissues. These peptides can also exert anticancer effects through multiple pathways, including the induction of cancer cell apoptosis, inhibition of proliferation, and regulation of the tumor microenvironment, demonstrating their ability to act on multiple targets. Additionally, in terms of drug toxicity, ACPs have significantly lower toxicity than conventional chemotherapy with minimal side effects, such as immune dysfunction or gastrointestinal toxicity, making them an effective alternative for patients who cannot tolerate traditional therapies [5]. Some naturally derived ACPs have been successfully extracted from food proteins through enzymatic hydrolysis. By selecting specific proteases and reaction parameters, the length and sequence of the peptides can be precisely controlled, making them suitable for large‐scale industrial production. Lin et al. [6] used trypsin, papain, bromelain, and alkaline proteases to hydrolyze *Enteromorpha prolifera* and isolated active ACPs. Among all the enzymatic products, the heptapeptide generated by trypsin hydrolysis exhibited the strongest anticancer activity. Zhang and Mu [7] studied the inhibitory effects of sweet potato protein hydrolysates prepared using six proteases on HT‐29 colon cancer cells. Among the fractions with molecular weights <3 kDa, the fraction with the strongest antiproliferative effect was identified. The fraction induced G2/M cell cycle arrest in HT‐29 cells, increased p21 and Bax expression, decreased Bcl‐2 expression, and activated caspase‐3, thereby inducing apoptosis. Sah et al. [8] conducted a comprehensive review of ACPs, generated during the enzymatic hydrolysis of milk proteins, highlighting the presence of bioactive peptides with anticancer potential in casein and whey proteins. Their findings underscore the promising application of food‐derived ACPs in the development of natural anticancer agents and functional dairy‐based products.

Although ACPs from natural sources can be efficiently obtained through enzymatic hydrolysis, identifying and screening these peptides from protein fragments still presents significant challenges. Traditional peptide identification methods rely on high‐performance liquid chromatography (HPLC) and mass spectrometry (MS), which can provide precise peptide mass spectra [9]. However, the large volume of data generated by MS makes it difficult for traditional screening methods to achieve rapid mining and accurate localization. The efficient, accurate, and rapid screening of peptides with anticancer activity has become a key bottleneck in developing peptide‐based drugs. In recent years, artificial intelligence (AI), with its efficient data processing capabilities, has gradually become an emerging tool for rapid discovery of bioactive peptides [10]. AI can learn the relationship between many peptide sequences and their biological activities in peptide screening to construct predictive models, identifying peptides with potential anticancer activity. Based on machine learning (ML) and deep learning algorithms, AI can analyze and process large‐scale peptide sequence data to extract patterns between peptide structural features and anticancer activity, thereby providing an efficient pathway for the rapid discovery of peptides [11]. This screening method enables the identification of ACPs, enhancing the efficiency of their discovery [12]. Ma et al. [13] applied deep learning algorithms to identify 40 potential ACPs from the gut microbiome, 39 of which exhibited inhibitory effects on at least one cancer cell line and were significantly different from known ACPs. This study improved the efficiency of ACP screening and opened new directions for the application of AI in cancer treatment.

AI provides new possibilities for large‐scale screening of ACPs, but current AI‐based ACP models primarily lack interpretability analysis and wet‐laboratory validation. Interpretability analysis in AI models helps decode the biological significance and reliability of the prediction results. The absence of clear interpretability often limits the biological foundation and credibility of ACP prediction models. Furthermore, while AI can enhance the accuracy of ACP discovery, the precision is not 100%, and the screening results still require validation through wet‐laboratory experiments. Wet‐laboratory experiments can reveal potential issues that AI models may fail to identify, such as peptide stability, toxicity, and interactions with other biomolecules. Unfortunately, there is limited research on the effective integration of interpretability analysis and wet‐laboratory validation into AI‐based ACP screening frameworks. To address this challenge, we systematically collected and integrated 68 ACP models (Table S1), summarizing their datasets, algorithms, performance metrics, and applications. We analyzed the limitations of current ACP prediction models based on the characteristics of these 68 models, focusing on the lack of interpretability analysis and wet‐laboratory validation. These limitations affect the reliability and effectiveness of practically applying these models. Meanwhile, we also propose integrating AI‐selected ACPs with multi‐omics, synthetic biology, amino acid modification technology, and nanodelivery technologies to enhance the targeting, stability, and efficacy of ACPs. Based on this, this review presents a comprehensive AI‐based ACP screening framework, covering the model construction process, interpretability analysis, wet‐laboratory validation, and integration with other biotechnologies. The framework aims to improve the efficiency, accuracy, and applicability of ACP screening, promoting the discovery and clinical application of novel ACPs.

## 2 AI‐BASED ACP PREDICTION TRAINING WORKFLOW

### Data set construction

**Figure 1:**
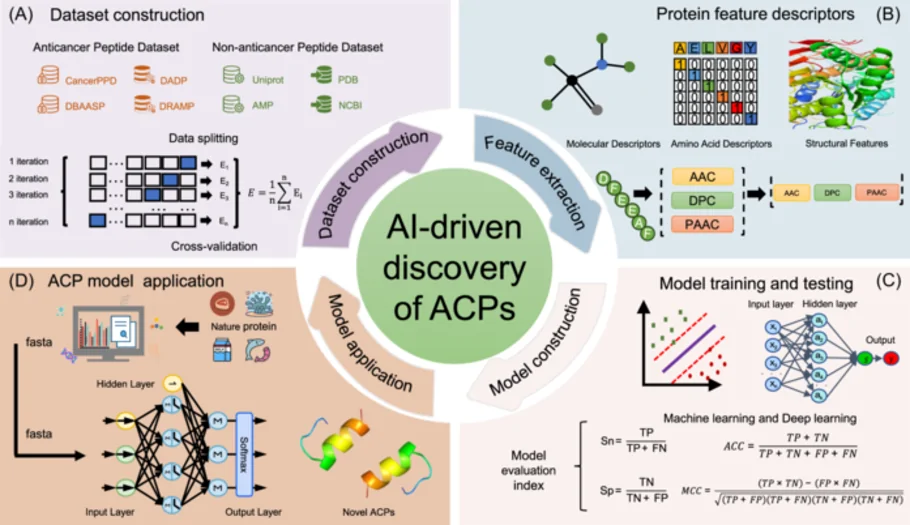
The establishment process of the anticancer peptide (ACP) AI model. (A) Construction of peptide datasets for anticancer and non‐anticancer peptides. (B) Extraction of protein features, including amino acid composition (AAC), dipeptide composition (DPC), pseudo‐amino acid composition (PAAC), and other molecular and structural descriptors. (C) Training and testing of the ML and deep learning models with evaluation metrics. (D) Application of the ACP model to identify novel ACPs from natural proteins.

Training datasets for ACP prediction models are extracted from public ACP databases, which provide the foundation for effective AI model development (Table S2). When building predictive models using ML, the quality and comprehensiveness of the data directly affect the accuracy and practicality of the models. A high‐quality public ACP database provides diverse data support for model training and offers new learning materials for model optimization and iterative updates (Figure 1A). For example, the Cancer Peptide Prediction Database (CancerPPD) is a public database dedicated to ACPs and their bioactivities. It includes the amino acid sequences of the peptides with important information about their effect on various cancer types [14]. In addition, ApInAPDB is a public ACP database that contains data on 818 known ACPs capable of inducing apoptosis in cancer cells. These peptide data are manually curated from research literature and other databases, ensuring the reliability and accuracy of the data. The database also provides detailed information about the peptide's functional types, target sites, half‐maximal inhibitory concentration (IC50) values, and other relevant data [15]. The data from these databases offer essential resources to build predictive models for ACP discovery.

In AI applications, models for ACP discovery typically include classification and regression models. Different models collect different types of data, ensuring that the models can focus on and analyze distinct features. The training of classification models includes both positive and negative datasets. The positive data set typically comes from public databases or is manually collected from research articles, while the negative data set generally consists of antimicrobial peptide datasets that lack anticancer activity or sequences from Uniprot that have not been reported [16]. Some models use antimicrobial peptides as negative training sets for ACP models because antimicrobial peptides and ACPs share similarities in amino acid sequences and structures, and both types of peptides exert their effects by interacting with cell membranes. Antimicrobial peptides primarily target the cell membranes of microorganisms, while ACPs act on cancer cell membranes. This similarity helps the model learn how to differentiate peptides with anticancer activity from those with only antimicrobial activity. Additionally, the antimicrobial peptide database is relatively well‐established and contains a large amount of data, providing more samples for the negative data set, ensuring diversity and completeness, and thus enhancing the model's generalization ability. The construction of the positive data set for classification models relies on collecting sequence data of ACPs. By analyzing these sequences, the model can learn the key features distinguishing ACPs from non‐ACPs and identify the potential anticancer activity of different peptides. Furthermore, the three‐dimensional structure of the peptide plays a key role in its bioactivity, directly influencing its ability to bind to target proteins, binding mode, and mechanism of action. By training classification models, researchers can mine peptides with potential anticancer activities on a large scale. Regression models typically do not require a negative data set. The data set collects quantitative indicators related to anticancer effects, such as inhibition rate, IC50, and other parameters. Regression models can accurately predict the anticancer activity of peptides by collecting and organizing these data. In addition to the bioactivity data, experimental validation results are an indispensable part of regression models. In vitro and in vivo experimental data, such as cell proliferation inhibition rates and tumor reduction effects, provide real‐world assessments of peptide effectiveness [17]. However, there has been relatively little research on ACP prediction based on regression models, with most studies focusing on classification models. This is likely because the bioactivity data for ACPs in public databases are relatively limited, and the effects of different peptides vary significantly for different cancers, making it challenging to construct regression model training datasets.

### Feature extraction

Feature extraction serves as AI‐based prediction and screening of ACPs. As systems cannot directly recognize the amino acid composition of peptides, specific algorithms are required to convert amino acid sequences into numerical feature representations. These features typically fall into three categories: molecular, amino acid, and structural features (Figure 1B). Among these, the study of molecular descriptors is relatively less developed, whereas amino acid descriptors are widely used, and structural features are gradually becoming the focus of peptide feature extraction.

#### Molecular descriptors

The molecular descriptors of peptides are used to quantify their physicochemical properties and convert them into numerical features that can be processed using ML algorithms. Symbolic representations of molecular structures are commonly used to achieve this transformation, such as a Simplified Molecular Input Line Entry System (SMILES) format that converts peptide sequences into chemical structures for easier computational processing and analysis. In the SMILES format, each amino acid residue and peptide chain connection can be represented as a sequence of characteristics, allowing the chemical structure of the peptide to be directly converted into quantifiable molecular descriptors [18]. RDKit is an open‐source toolkit widely used in cheminformatics that can parse SMILES representations, generate molecular structure diagrams, and compute various molecular descriptors. RDKit can extract a variety of molecular descriptors from SMILES representations, which then serve as input features for AI models [19].

Although molecular descriptors provide basic chemical information about peptides, they typically reflect only their physicochemical properties, such as bond lengths, highest occupied molecular orbitals (HOMO), and lowest unoccupied molecular orbitals (LUMO) within the molecular structure. However, these indicators primarily focus on the static structure and electronic characteristics of the molecule, and cannot fully capture the biological behavior and activity of peptides. The biological activity of peptides is closely related to their complex molecular interactions, particularly with their target proteins. Factors such as the peptide's secondary structure, three‐dimensional folding, amino acid sequence arrangement, and hydrophilic/hydrophobic properties significantly influence their binding affinity and stability with the target. These aspects are not typically captured by traditional molecular descriptors. As a result, the application of molecular descriptors has been focused on screening anticancer molecules and drug design. In ACP research, relying solely on molecular descriptors may not sufficiently capture the biological characteristics of peptides, particularly their ability to bind to target proteins, stability, and efficacy in vivo. For example, Balaji et al. [20] obtained data on 10,900 active and inactive small anticancer molecules from the NCBI database, represented in the SMILES format. Each compound was calculated to obtain 1446 one‐dimensional and two‐dimensional molecular descriptors. To optimize the feature selection process, they used the VarianceThreshold method in Scikit‐learn for dimensionality reduction and trained an anticancer molecule prediction model, MLASM. In comparison, molecular descriptors have been applied to study other bioactive peptides, such as umami peptides and antimicrobial compounds. These peptides are typically represented in SMILES format, and their molecular‐level features are extracted through molecular descriptors for quantitative analysis and feature characterization. Cui et al. [21] used RDKit to extract the molecular descriptors of umami peptides, obtained descriptor features for 278 peptides, and trained the Umami_YYDS model based on these features. PmxPred was developed to accelerate the discovery of polymyxin compounds for multidrug‐resistant Gram‐negative bacteria by using RDKit descriptors to represent molecules and residues in combination with ensemble learning models for predicting antimicrobial activity [22].

#### Amino acid descriptors

Amino acid descriptors are important for the feature extraction of ACPs (Table S3). This feature extraction method can accurately reflect the semantic information, chemical composition, structural characteristics, and the arrangement and interaction of amino acids, helping AI models gain a deeper understanding of the biological activity of peptides. Amino acid descriptors are also the most commonly used feature extraction method in peptide models today. To better capture these features, commonly used amino acid descriptors include Amino Acid Composition (AAC), Dipeptide Composition (DPC), Tripeptide Composition (TPC), Pseudo Amino Acid Composition (PseAAC), and Amino Acid index (AAindex). These methods quantify the frequency, relative position, and interaction relationships of amino acids and are widely used in feature extraction and prediction model construction for ACPs [23]. For example, Garai et al. [24] used multiple amino acid descriptors, including AAC, to construct compositional features and train an LGBM‐ACP model. The prediction accuracy of this model was 97.52%, and molecular docking analysis validated the strong affinity of the predicted ACPs for multiple drug targets. In addition to amino acid composition, amino acid position and pair features can more precisely reflect the arrangement and interactions of amino acids within the peptide. For example, BLOSUM62 and Position‐specific scoring matrix (PSSM) are commonly used tools for quantifying amino acid substitution patterns and their relative importance at different positions within protein sequences. BLOSUM62 helps capture the position dependence of amino acids in the peptide sequence by evaluating the likelihood of amino acid substitutions, whereas PSSM can reveal the potential impact of the position of specific amino acids on the peptide's function and structure [25, 26]. BLOSUM62 and PSSM, when combined with other amino acid descriptors, can further improve the prediction accuracy of the models. The ACP‐ML model extracts features from peptide sequences using methods, such as DPC, PseAAC, Composition/Transition/Distribution (CTD), and CS‐Pse‐PSSM, and achieves an accuracy rate of over 90% [27]. Amino acid descriptors are not limited to traditional compositional and positional features; they can be represented using one‐hot encoding and protein language models. One‐hot encoding is a basic encoding method that converts amino acid sequences into fixed‐length vectors, making it easier for AI models to process. Liu et al. [28] combined one‐hot vectors with an ensemble learning framework to train a model called AntiMF, which captured information from different dimensions, thereby improving the representation and prediction accuracy of ACPs. However, one‐hot encoding does not capture the sequential relationships and contextual information between amino acids, restricting its expression ability in more complex tasks. Protein language models have gradually become the focus of research to address this issue. Protein language models, such as ProtBERT, ProGen, and ESM, based on Transformer and Bidirectional encoder representation converter (BERT) architectures, are pretrained on large‐scale protein sequence data and effectively capture both the contextual dependencies and evolutionary patterns within amino acid sequences [29]. The embedding vectors generated by these models include the features of individual amino acids and reflect their positions and functional relevance within the sequence, offering richer representations [30]. Furthermore, combining protein language models with conventional amino acid descriptors can significantly improve the model performance. For example, Niu et al. [31] built a comprehensive ACP feature matrix by combining natural language processing and PseAAC technology to extract the typical first and second structural properties, demonstrating excellent predictive ability in ACP recognition.

#### Structural features

Owing to its rapid development, AI has begun to assist in the structural analysis in bioinformatics, enhancing the interpretation of complex biological data. Advanced AI tools such as AlphaFold3, trRosetta, and pep‐fold have significantly improved the accuracy of peptide and protein 3D structure prediction [32, 33, 34]. These tools use deep learning methods to train large datasets of protein sequences and structures, enabling the efficient and accurate prediction of peptide molecular 3D structures and providing more reliable structural information. These structural features are suitable for training models of biomolecules with complex structures, as they help the model to effectively capture the three‐dimensional spatial arrangement of peptides and the intramolecular interactions. For example, TP‐LMMSG utilizes the trRosetta model to construct basic peptide graphs based on peptide sequences, which are then used to predict amino acid connectivity and derive the 3D structure of peptides as input features for the models, thereby improving the prediction accuracy for various types of bioactive peptides [35].

Although structural features are highly valuable for model prediction, obtaining these ACP structural features in large quantities is challenging, primarily due to limitations in computational resources. The prediction of peptide three‐dimensional structures involves complex protein structure prediction techniques, which require substantial computational resources and time. This makes it challenging to perform predictions on large‐scale datasets. Generating batch structural features of peptides and target proteins requires high‐performance computing platforms to ensure the accuracy and efficiency of the predictions. As a result, these structural features are less commonly applied in ACP prediction but are more frequently used in the molecular simulation stage following AI screening. In the molecular simulation stage, these AI tools provide key input files for molecular docking and molecular dynamics simulations. Molecular simulations revealed the key residues and interaction mechanisms during the binding process between peptides and target proteins, offering molecular‐level references for the stability of peptide functionalization. Molecular docking was used to simulate the binding sites of peptides to target proteins and optimize peptide design to enhance binding specificity and stability. Molecular dynamics simulations further expand our understanding of the interactions between ACPs, target proteins, and cancer cell membranes. Simulating the dynamic behavior of peptide–protein complexes and peptide–cancer cell membrane interactions makes it possible to analyze the binding stability, conformational changes, and details of key interactions. Panjeta et al. [36] used molecular dynamics to study the potential of human defensin five combined with 5‐fluorouracil (5‐FU) in colon cancer treatment, especially against 5‐FU‐resistant colon tumor cells. The simulation results indicated that the peptides and chemotherapeutic drugs could bind to altered membrane fluidity levels in cancer cells, enhancing their anticancer effects. Sevim and Altuntas [37] performed molecular dynamics simulations to investigate how cysteine substitution affects disulfide bonds and the overall thermal stability of mutated peptides. These findings revealed that the redesigned ACPs maintained their helical structure and thermal stability even at high temperatures, indicating their potential as effective antibacterial alternatives.

### Model construction

ACP prediction models primarily include traditional ML and deep learning models, with training strategies like cross‐validation employed to ensure their generalizability and stability. After training, the model performance was evaluated using various metrics such as accuracy (ACC), area under the receiver operating characteristic curve (AUC), Matthews correlation coefficient (MCC), sensitivity (Sn), and specificity (Sp), ultimately selecting the best‐performing ACP prediction model (Figure 1C).

#### Traditional machine learning

**Figure 2:**
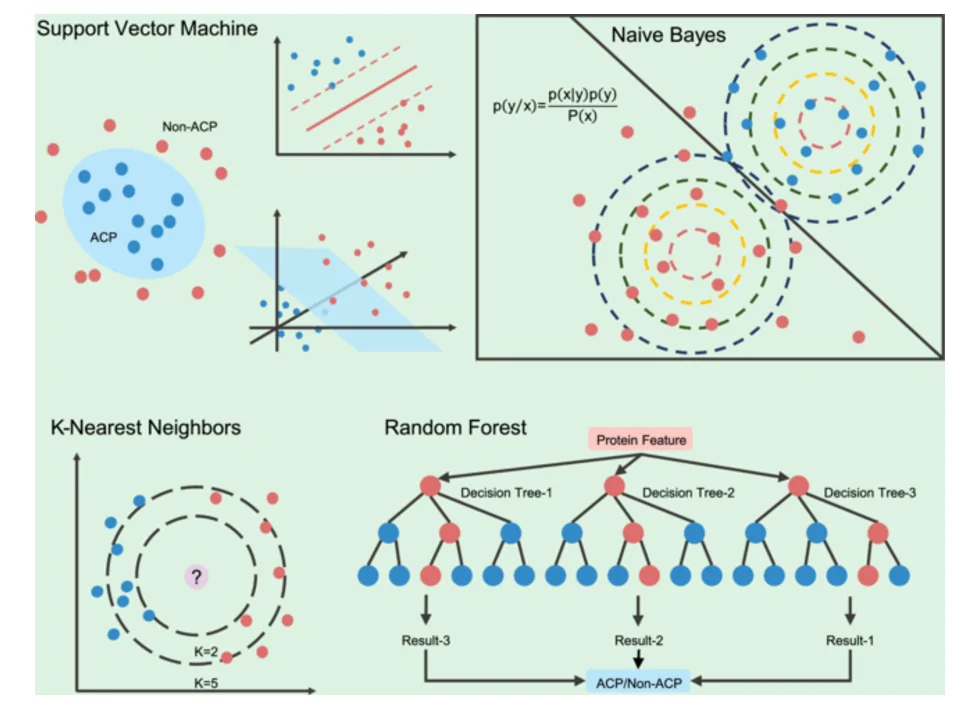
Examples of commonly used traditional ML algorithms for anticancer peptide (ACP) models, Support Vector Machine, Random Forest, K‐Nearest Neighbors, and Naive Bayes. Blue circles represent ACP, and red circles represent Non‐ACP. In the Support Vector Machine diagram, the lines denote the decision boundary separating ACP and Non‐ACP. In the K‐Nearest Neighbors and Naive Bayes diagrams, dashed circles show decision boundaries based on distance and probability distribution. In the Random Forest diagram, the lines in decision trees represent the feature‐based splits distinguishing ACP from Non‐ACP.

Traditional ML models have been widely applied in predicting ACPs. These models construct feature matrices from amino acid descriptors and train the model based on traditional ML algorithms. Traditional ML methods include Support Vector Machine (SVM), Random Forest (RF), K‐Nearest Neighbor (KNN), Naive Bayes (NB), and others (Figure 2). Several ACP prediction models have adopted ensemble learning methods in recent years. By combining multiple traditional ML models, ensemble learning leverages the advantages of different models to compensate for the limitations of the individual models. The ensemble model reduces variance and bias by training different sub‐models multiple times and performing weighted averaging or voting on the prediction results, thereby enhancing the robustness of the model and improving its generalization when faced with complex and diverse data.

#### Support vector machine

SVM performs well in handling high‐dimensional data and has strong resistance to overfitting. Its performance mainly depends on the selection and tuning of the kernel function and key parameters. The commonly used radial basis function (RBF) kernel can adapt to different data distributions, while the regularization parameter affects model complexity and generalization ability. These settings are typically optimized through cross‐validation to ensure the best performance across different datasets [38]. Wan et al. [39] constructed a feature matrix using AAC, N5C5, K‐space, and PSSM based on the SVM framework combined with Sequence Minimal Optimization to optimize the performance of the ML model to effectively distinguish ACPs from antimicrobial peptides. The iDACP model, built on the LIBSVM framework, applies a two‐step ML approach to identify and classify ACPs. It analyzes the amino acid composition at the N‐terminal and C‐terminal of peptides, classifying ACPs into three main subtypes based on the distribution of positively charged residues. By integrating sequence features with physicochemical properties, iDACP enhances predictive accuracy [40]. Ge et al. [41] developed an EnACP model using the SVM algorithm that combines multiple feature representations, including sequence composition, sequence order, and physicochemical properties, to efficiently and accurately identify ACPs.

#### Random forest

RF is an ensemble method using multiple decision trees to improve accuracy and robustness. Its performance depends on tree number, depth, sample split, and feature selection randomness. More trees enhance stability but increase cost; excessive depth or too few splits may overfit, while the opposite may underfit [42]. The ACPred model uses the RF framework to build the prediction model and differentiate ACPs from non‐ACPs by evaluating the importance of various features. By applying the RF method, ACPred identified key features influencing ACP activity, including hydrophobic residues, α‐helix structure, and β‐sheet formations. Following cross‐validation, the model achieved an overall accuracy of 95.61% in recognizing ACPs [43]. The RFaaindexACP model uses the RF algorithm to train and calculate the feature importance to identify the key physicochemical properties of amino acids associated with ACPs. The model ultimately identified 19 key physicochemical properties of amino acids that could be used to predict the anticancer potential of peptide sequences [44]. Deng et al. [45] developed a two‐stage prediction model, ACP‐MLC, to identify ACPs and classify their functions. In the first layer, the model uses the RF algorithm to predict whether a given peptide sequence is an ACP sequence. In the second layer, the model adopts a binary correlation algorithm to predict the tissue types targeted by the sequence.

#### *K*‐Nearest neighbor

KNN classifies samples by calculating the distance between them, with its performance mainly affected by the *K* value and the choice of distance metric. A small *K* value can lead to overfitting, while a large *K* value may cause underfitting. In addition, the choice of distance metric has a significant impact on prediction performance; common metrics include Euclidean, Manhattan, and Minkowski distances, and selecting an appropriate metric can improve prediction accuracy [46, 47]. Alsanea et al. [48] proposed an ensemble classifier that combined SVM, RF, and KNN algorithms to precisely identify ACPs. This ensemble method improves prediction accuracy and robustness by integrating the advantages of multiple classifiers. The experimental results for the benchmark and independent datasets showed 97.09% and 98.25% accuracy rates, respectively.

#### Naive Bayes

NB is a probabilistic classification method based on Bayes theorem, which classifies by calculating the probability of features under each class and combining it with the prior probability. Its performance is affected by the smoothing parameter, which is used to avoid the zero‐probability problem. In addition, different NB variants are suitable for different data distributions, and selecting the appropriate variant along with tuning the smoothing parameter during model training can further improve classification performance [49]. Lv et al. [50] compared several traditional ML methods for ACP classification when training the iACP‐DRLF model and found that NB performed poorly. This may be due to the assumption of feature independence, which does not align with the complexity of the amino acid distribution and structural features in peptide sequences. Therefore, NB is often combined with other ML algorithms to enhance the prediction performance. Danish et al. [51] developed an ensemble model by combining SVM, RF, and NB algorithms. The model integrates four peptide encoding methods—AAC, DPC, TPC, and enhanced pseudo‐amino acid composition (EPseAAC)—to present peptide features more effectively. After testing, the ensemble model achieved accuracy rates of 97.56% and 95.00% for the benchmark and independent datasets, respectively.

#### Deep learning

Deep learning models have also been widely applied to screening and predicting ACPs, including Artificial Neural Networks (ANN), Recurrent Neural Networks (RNN), Convolutional Neural Networks (CNN), Graph Neural Networks (GNN), and hybrid models. These deep learning models are suitable for large datasets and can automatically extract complex features from large amount of data, thereby improving model performance. When the data volume is large, traditional ML models may face challenges in feature engineering and overfitting, whereas deep learning models, with their powerful feature learning capabilities and automatic optimization mechanisms, are better equipped to handle complex, high‐dimensional data and achieve higher prediction accuracy. Hybrid models have become a trend in deep learning, combining ANN, RNN, CNN, and GNN to fully utilize the strengths of each model and improve prediction accuracy. In addition, incorporating attention mechanisms into hybrid models allows the model to focus on key feature regions and critical amino acid residues within ACP sequences, enhancing prediction performance and interpretability.

#### Artificial neural networks

**Figure 3:**
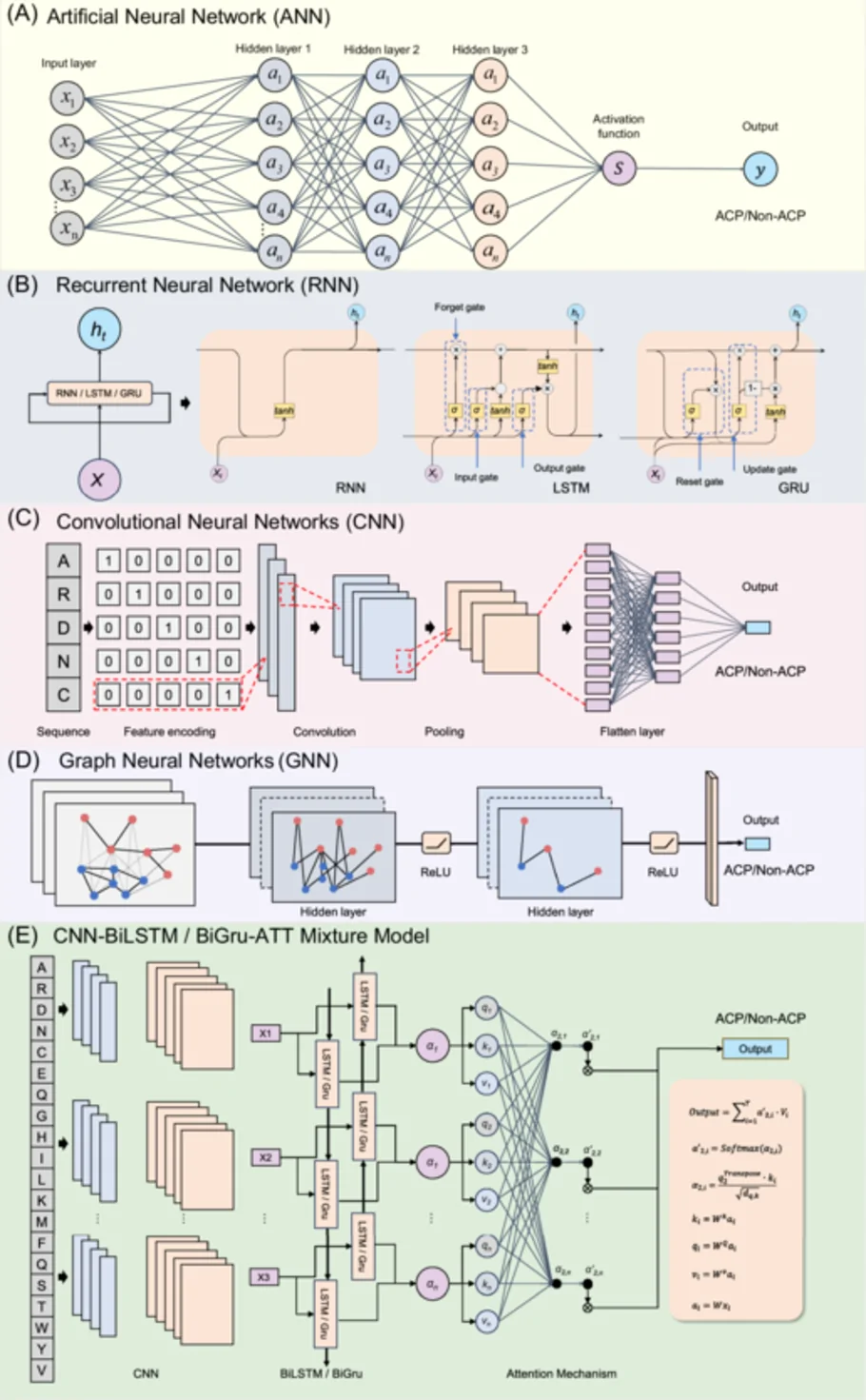
Examples of commonly used deep learning algorithms for ACP models, where circles represent neurons and lines represent connections or weights that transmit information between neurons, including (A) Artificial Neural Network, (B) Recurrent Neural Network, (C) Convolutional Neural Network, (D) Graph Neural Network, and (E) Hybrid models. ACP, anticancer peptide.

ANNs are ML models that simulate biological neural systems, consisting of input, hidden, and output layers, and learn by adjusting connection weights (Figure 3A). Their performance is influenced by factors such as network structure, activation function, and learning rate: the number of hidden layers and neurons per layer determines model complexity; activation functions like Sigmoid, ReLU, and Tanh introduce nonlinear characteristics; and the learning rate controls the training step size, which should be set appropriately to balance stability and efficiency [52]. Kaushik et al. [53] developed an A‐CaMP model using an ANN that quickly identified anticancer and antimicrobial peptides. Using clinical data, A‐CaMP can provide detailed information on wild‐type and mutant sequences and peptides, supporting cancer and bacterial infection treatments. ENNAACT employs a neural network framework and deep learning techniques to develop a sequence‐based ACP activity classifier. The performance of this model was comparable to that of the best classifiers in the field, achieving an accuracy of 98.3% after cross‐validation [54].

#### Recurrent neural networks

RNNs are deep learning models well suited to sequential data, with applications in time‐series analysis, natural language processing, and biosequence analysis (Figure 3B). However, RNNs often suffer from vanishing or exploding gradients on long sequences; long‐range dependencies may be forgotten, leading to ineffective learning and limited performance [55]. To address this, variants such as long short‐term memory (LSTM) and gated recurrent units (GRU) were introduced, among which LSTM is more commonly used in ACP deep‐learning models [56]. ACP‐DL combines the LSTM algorithm with a Binary Profile Feature and K‐mer Sparse Matrix for efficient feature representation. Cross‐validation tests showed that ACP‐DL outperformed the other methods on benchmark datasets [57]. Furthermore, Bidirectional LSTM (BiLSTM), which computes the sequence in both the forward and reverse directions, is particularly suitable for peptide sequence analysis, allowing the model to capture both forward and reverse contextual information. ACP‐DRL employs a BiLSTM framework and applies protein‐language models to ACP identification. Using BiLSTM to extract amino acid sequence features, ACP‐DRL overcomes the sequence length limitation and eliminates the need for manual feature extraction, thereby advancing deep learning in ACP recognition [58].

#### Convolutional neural networks

CNNs consist of convolution and pooling modules that automatically extract local features via sliding windows and reduce dimensionality, thereby lowering computational cost and the risk of overfitting. Through repeated convolution and pooling, CNNs progressively derive higher‐level semantic representations from low‐level features such as edges and textures (Figure 3C). The ACP‐2DCNN uses a 2D‐CNN structure in combination with the Dipeptide Deviation from Expected Mean (DDE) method to extract features [59]. In addition to a 2D‐CNN, a 1D‐CNN structure is also suitable for one‐dimensional peptide sequence features. When processing peptide sequences or other one‐dimensional sequence data, a 1D‐CNN effectively extracts local amino acid features within the peptide sequence. Compared with traditional sequence models, 1D‐CNN exhibits a strong feature‐learning ability in peptide sequence analysis and automatically learns effective representations from large‐scale datasets, thus reducing the need for manual feature engineering [60]. Sun et al. [61] trained a novel ACP prediction model, AI4ACP, which integrates a new peptide sequence encoding method, PC6, representing six physicochemical properties for each amino acid. In addition, AI4ACP incorporates the CNN model framework and employs five‐fold cross‐validation, achieving an accuracy of 0.89. Aziz et al. [62] used binary encoding for feature extraction and trained an ACP model, iACP‐MultiCNN, using a multichannel CNN. They developed a web server for their model. mACPpred 2.0 constructed a feature matrix by combining traditional feature descriptors and advanced embeddings based on pre‐trained natural language processing and employed stacked deep learning (SDL) methods with 1D‐CNN blocks for model training. Through rigorous cross‐validation and independent testing, mACPpred 2.0 outperformed its previous version, mACPpred [63].

#### Graph neural networks

GNNs propagate information through inter‐node connections, effectively capturing node dependencies and structural characteristics in graphs. Compared with traditional sequence models, GNNs better characterize topological and latent spatial structures and are more suitable for modeling structural features of peptides (Figure 3D) [64, 65]. ACPScanner combines sequence information, physicochemical properties, structural information, and protein semantic information to construct an ACP feature space and combines LightGBM and GNN for model training. LightGBM processes the input through SVM‐Prot, NetSurfP‐3.0 features, and average pooling from ESM‐1b embeddings, which use the feature set of each amino acid residue as a node attribute in the graph and employ contact graphs derived from the predicted coordinates as edges, improving the competitiveness of the model [66]. Graph Convolutional Network (GCN) is a special variant of GNN, with the main difference being in the feature aggregation method. Whereas traditional GNNs rely on recursive or cyclic propagation mechanisms to update node representations, GCN uses graph convolution operations, utilizing the graph's Laplacian matrix to perform efficient feature aggregation, thereby improving its ability to capture graph structural information. Rao et al. [67] used one‐hot encoding to extract the features of ACPs and transformed the ACP prediction task into a graph classification problem. Each peptide sample is represented as a graph structure, and the GCN automatically extracts and learns its features to construct an ACP‐GCN prediction model.

#### Hybrid models

Hybrid models are becoming increasingly effective in predicting ACPs, with many of them combining CNN and RNN. CNN provides strong local feature‐learning capabilities, whereas RNNs capture long‐term dependencies in sequences, enhancing the overall modeling capability for peptide sequences (Figure 3E). Yuan et al. [68] trained an ACP‐OPE using BiLSTM and CNN with ML module built using the LightGBM framework. The final model was built by integrating the classification results from the three models along three paths using a voting mechanism to optimize the prediction performance for ACPs. Additionally, combining CNN, RNN, and GNN with attention mechanisms enhanced model performance. In attention mechanisms, Q (query), K (key), and V (value) are the three important vector concepts where the query typically represents the current input feature, the key represents reference information related to the input feature, and the value represents the actual value information. The attention mechanism calculates the matching score between Q and K using a dot product and then uses the softmax function to convert these scores into a probability distribution, which is used as a weight for the corresponding V [69]. Finally, through weighted summation, the model generates a feature representation focusing on key information. This mechanism enables the model to adjust its attention to different input features dynamically, thereby effectively reducing interference from irrelevant information. The ACPred‐BMF model uses the quantitative and qualitative properties of amino acids and binary features to numerically represent peptide sequences by combining BiLSTM and attention mechanisms to enhance the feature extraction capability of the model [70]. He et al. [71] trained ACPred‐LAF, using a multi‐head attention mechanism and fully connected neural networks, incorporating multi‐perception and multi‐scale embedding techniques to automatically learn and extract ACP sequence features. This method overcomes the limitations of experience‐based feature engineering and improves the representation and adaptability of models. When a GNN is combined with the attention mechanism, it becomes a Graph Attention Network (GAT). In the GAT, each node has adaptive weights during information aggregation, enabling the model to precisely capture the importance of different neighbors in the learning process [72]. The TP‐LMMSG, a new graph deep‐learning model based on GAT, uses the three‐dimensional structure prediction tool trRosetta to construct a basic peptide graph from the peptide sequence, thereby predicting amino acid connectivity and fully utilizing the structural information of the peptide molecules. Moreover, optimizing the TP‐LMMSG significantly improves the prediction accuracy and reduces the preprocessing time by more than seven times compared to previous graph‐learning models, greatly enhancing the prediction efficiency.

### Application of ACP models

The application of ACP models relies on the development of omics technologies, including peptide identification and prediction in metagenomics, transcriptomics, and proteomics. These technologies enable users to obtain large amounts of peptide sequence data, providing valuable resources for the discovery of ACPs. In combination with instruments such as liquid chromatography‐MS (LC‐MS), the analysis of low‐abundance peptides, which were previously difficult to analyze, can now be performed with high efficiency. This combination enables the accurate separation, identification, and quantification of peptides, facilitating precise identification of peptide sequences within complex protein fragments [73, 74]. After matching and identifying these peptide sequences with protein databases, they undergo preprocessing steps such as redundancy removal and standardization, and are ultimately stored in formats like FASTA [75]. These data are fed into predictive models, where, following data preprocessing and feature extraction, ACP mining is carried out (Figure 1D).

Currently, some ACPs predicted through AI screening have been successfully identified from food‐derived proteins and have been experimentally validated for their anticancer activity. Food‐derived proteins, widely available and sustainable sources of biomolecules, have gained attention in recent years because of their potential biological activities, making them essential raw materials for developing functional foods. Through enzymatic hydrolysis, these proteins can be targeted and broken down into shorter peptide sequences, which not only have higher bioavailability but may also exhibit antimicrobial, antioxidant, and anticancer activities. Chantawannakul et al. [76] used three ML models to screen new ACP candidates from the proteolytic products of *Cordyceps militaris* (CM) protein hydrolysates. This screening process identified several ACP candidates from CM that could serve as potential alternative or adjunctive treatments for colorectal cancer, reducing the dependence on conventional chemotherapy treatments. Lerksuthirat et al. [77] identified two potential ACPs, ALA‐A1, and ALA‐A2, from α‐lactalbumin using in vitro screening combined with three ML models. In vitro experimental results showed that ALA‐A2 exhibited specific cytotoxicity against A549 lung cancer cells without hemolytic effects. ALA‐A2 also demonstrated good cell penetration ability and promoted lung cancer cell death by inducing autophagy. In addition, other naturally derived ACP vaccines have also been discovered through AI. Some ACP models, by analyzing immune‐regulating peptides from arthropods, have identified short peptide sequences with antimicrobial and anticancer activities. Further molecular docking studies have determined the interaction of these peptides with human cell surface TLR receptors, demonstrating the potential of peptides as cancer immunoadjuvants [78]. By integrating a high‐quality data set of 16,349 ncRNA‐derived micropeptide sequences and evaluating the expression of micropeptides with high‐throughput MS data from breast cancer subtypes, de Azevedo et al. [79] used multiple ML tools to predict the functions, cellular localization, and physicochemical properties of micropeptides, confirming their potential for anticancer, anti‐inflammatory, and antiangiogenesis activities. Scieuzo et al. [80] analyzed the transcriptome of the red palm weevil (*Rhynchophorus ferrugineus*) and predicted its antimicrobial activity. Using multiple ML algorithms derived from the CAMPR3 database, they analyzed sequences presumed to encode antimicrobial peptides, and by applying tools like iACP, AVPpred, and Antifp, they predicted the anticancer, antiviral, and antifungal activities of these candidates, further mining the peptide resource library of the red palm weevil. In summary, the application of AI models in the discovery of natural ACPs is still in its early stages, with advancements in model interpretability algorithms, it is expected that more research based on modern omics technologies and AI methods will emerge in the future, enabling the efficient discovery of new ACPs from natural proteins, and providing more efficient and precise strategies for cancer treatment based on natural substances.

It is important to note that although AI has started to be applied in ACP screening, most models are still at the stage of model construction. This is partly due to the lack of interpretability analysis, which hinders the confidence in clinical applications. On the other hand, the absence of biological experimental validation may lead to poor model generalization. To enhance the application of AI in ACP screening, we will next discuss the limitations of AI models in ACP prediction and the areas for improvement.

## 3 LIMITATIONS AND IMPROVEMENT STRATEGY FOR AI‐BASED ACP PREDICTION MODELS

Based on the 68 ACP models summarized by us (Table S1), we analyzed the strengths and weaknesses of each model and discussed the limitations of AI in ACP applications from the perspectives of model bias, model interpretability, and biological validation. To address the limitations of the model, we have proposed solutions and recommendations for each issue, with the aim of improving the model's generalization ability and clinical applicability.

### Model bias

#### Training data set bias

We found substantial differences in the size of the training datasets among the 68 ACP models that we analyzed (Table S1), ranging from a few hundred to several thousand samples. Interestingly, some models also exhibit an imbalance in the distribution of positive and negative samples, with a significant difference in the number of positive and negative samples. These imbalances can lead to a model favoring one class during training, affecting the generalization ability of the model. Most models classify all peptides with anticancer activity as positive and primarily focus on predicting whether a peptide shows anticancer activity. Some models also predict the type of ACP in addition to predicting whether a peptide is an anticancer agent. These multiclass models assign peptides to different training sets based on cancer types, which can easily lead to large differences in the number of samples across the training sets, affecting the model's prediction performance on different cancer types. Such as, ACPScanner predicts ACPs and their specific anticancer function type. The training set covers nine types of cancer; however, the data set size widely varies among cancer types, ranging from 23 to 129 samples. The model may thus have poorer generalization ability and less accurate predictions for cancers with fewer samples in the datasets. For the issue of data imbalance, efforts still prioritize collecting datasets with equal sample sizes. However, considering that some types of ACP samples are limited, efforts also involve the use of technical support such as Cluster Database at High Identity with Tolerance (CD‐HIT) for redundancy removal and data augmentation methods. CD‐HIT is used to remove redundant or similar sequences by setting a similarity threshold, which reduces the amount of redundant data. The CD‐HIT threshold is typically set to 0.9 or 0.8 to remove similar sequences according to the 68 models we reviewed. This approach ensures that the model is not affected by redundant data during training, strengthening the ability of the model to learn diverse samples and enhancing its generalization ability. In addition, more samples are generated using data augmentation by transforming existing samples, such as through rotation, translation, scaling, and cropping. Data augmentation increases not only the training data volume but also the ability of the model to adapt to different data features, helping the model learn more patterns and reducing the risk of overfitting [81, 82]. Data augmentation techniques are used to address the issues associated with small sample sizes and class imbalances for certain bioactive peptide samples. BPFun is a deep‐learning‐based multiclass model for predicting seven types of functional peptides that uses data augmentation to address the problem of missing small‐sample categories [83]. Data augmentation techniques, despite their effectiveness in addressing data bias and enhancing performance, have rarely been applied with ACP models. However, with the increasing exploration of ACP resources, it is believed that data augmentation techniques can effectively address the imbalance issue in the training sets of multiclass ACP models, improving the performance of multiclass prediction models.

#### Model overfitting

Since most of the 68 models have not been validated under wet‐laboratory conditions and lack testing for generalization across different cancer types or patient datasets, it is difficult to directly determine whether the models are overfitting based solely on model evaluation parameters. Relying solely on the performance on training datasets makes it difficult to fully assess the model effectiveness in real‐world applications. The development of new ACP models in the future will require comprehensive testing to ensure the models have good generalization ability. To minimize overfitting as much as possible during training, in addition to the potential data set bias that may lead to overfitting and insufficient generalization, the complexity of the model itself may also affect the model's generalization ability. Consequently, it is recommended to simplify the model structure by using dropout or applying regularization techniques, such as L1 or L2 regularization, to improve model generalization ability [84, 85]. MLACP 2.0 has a complex structure with 17 different feature encodings and seven different classifiers. Dropout is set to 0.5 in the model to reduce overfitting: the model randomly drops neurons to reduce the complexity, dependency, and overfitting of the model [86]. The dropout is set to 0.3 in ACPs‐ASSF to reduce overfitting, limit model complexity, and ensure generalization ability when used with diverse data [87]. In addition, Regularization is another method for effectively reducing model overfitting. Unlike Dropout, regularization is used to control model complexity by adding a penalty term to the loss function. Common regularization methods include L1 and L2 regularization. L1 regularization encourages sparse model weights, selecting the most relevant features; L2 regularization penalizes high weights, smoothing the weight distribution of the model and avoiding overfitting to the training data [88]. Such as, L2 regularization is used in ACP‐DA, which helps reduce the overfitting of the training data, allowing the model to focus on general patterns during the learning process and avoid overly relying on certain features [89].

In addition to reducing model complexity, the cross‐validation settings during feature processing are also an important method for reducing model overfitting. In cross‐validation, a data set is divided into multiple subsets, and multiple training and validation cycles are performed on different subsets. This method is used to evaluate the generalization ability of the model and prevents overfitting to a single training set, increasing model stability and reliability [90, 91, 92]. The common cross‐validation methods include k‐fold cross‐validation, leave‐one‐out cross‐validation, stratified k‐fold cross‐validation, and bootstrapping. Five‐fold cross‐validation is used in AntiCP 2.0 for training and testing the model. This method divides the data set into five subsets, using four subsets for training and the remaining one subset for validation. The process is repeated five times, with each subset used once as the validation set. The performance of the model is evaluated by aggregating the results from all five validations to select the most appropriate training model [93]. The ACP‐LSE model was used to perform 10‐ and 5‐fold cross‐validation on the ACP‐344 and ACP‐740 datasets, respectively, to ensure model stability [94]. It is important to note that cross‐validation increases the stability of a model using different subsets of data, but substantially increases the time required for training, especially for large fold numbers, as the training time exponentially increases with increasing fold number. Hence, the parameters and number of folds must be appropriately set based on the performance of the GPU to balance the accuracy and training efficiency of the model. Table S1 lists the training times required for different models on different devices to provide a reference for developing new ACP models on specific devices.

#### Inconsistency in benchmark test sets

The accuracy of the 68 models ranged from 75% to 99%. However, a model with 99% accuracy may not be the most suitable for predicting ACPs because the different test sets were used for the different models, preventing a fair comparison of model performance. The samples, feature distributions, and label categories of each test set differ, and the performance of one model on a data set may vary depending on how the data are partitioned. Relying solely on model accuracy does not allow for the identification of the most suitable model for predicting ACPs. Consequently, it is necessary to establish a comprehensive, accurate, and universally recognized test data set to ensure consistency and fairness when evaluating different models. Any established base test set must be fair, prevent data leakage, and ensure that peptide information from the test set is not improperly incorporated into the training set, so the model performance in real‐world applications can be accurately reflected [95]. At the same time, include testing on different cancer types or patient datasets as much as possible to evaluate the model's generalization ability. Interestingly, model test sets are inconsistent in areas other than ACP research; similar issues arise during model comparisons for nearly every new model developed for bioactive peptides. The use of dry metrics such as accuracy is insufficient for reflecting the actual performance of the model. Thus, wet‐laboratory experimental validation is required to ensure the reliability and effectiveness of model predictions, in addition to evaluating parameters such as ACC, AUC, and MCC.

### Model interpretability

#### Lack of model interpretability

**Figure 4:**
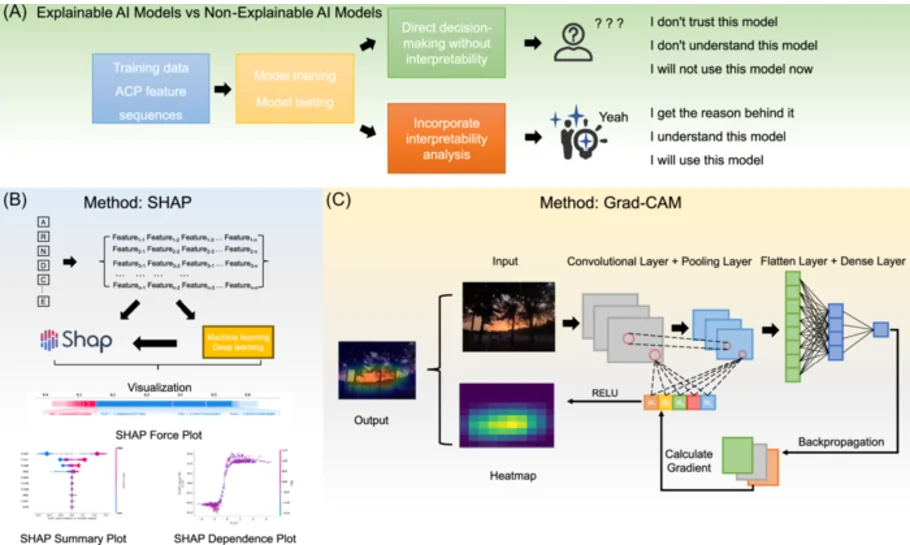
Schematic of AI interpretability analysis: SHAP and Grad‐CAM. (A) Comparison of explainable AI models and non‐explainable AI models. (B) SHAP method for explaining model predictions, with visualization methods including SHAP Force Plot, SHAP Summary Plot, and SHAP Dependence Plot. (C) Grad‐CAM method for visualizing the contributions of input features in deep learning models, with the process from input to output, demonstrating the use of gradients to generate heatmaps for interpretation.

In AI‐driven bioactive peptide prediction tasks, high model performance is often achieved at the cost of complex nonlinear structures, making the decision‐making processes difficult to interpret directly. This “black‐box” problem limits the usability and trustworthiness of AI in practical applications [96]. In Table S1, only 16 out of the 68 models have undergone interpretability analysis, accounting for less than a quarter, which may also be a reason for the limited application of these models. The application of AI models in clinical translation requires ensuring the reliability and reproducibility of the model, and black‐box models lacking interpretability are difficult to gain acceptance in the medical community. In medical applications, doctors and researchers must be able to understand the reasoning behind the model's predictions to trust the results in practical use (Figure 4A). For instance, in the development of novel anticancer drugs, only clear decision‐making foundations can ensure that the model‐predicted candidate peptides have clinical efficacy and avoid potential risks arising from incorrect model judgments. Interpretability analysis can reveal which features have the greatest impact on the model's predictions, helping to identify which peptide sequences might be potentially effective drugs and providing data support for clinical trial design. As clinical experimental data accumulates, interpretability analysis can feed back into the model, continuously optimizing the model's performance and prediction accuracy, ultimately facilitating the clinical translation of the model. Therefore, to improve the transparency of ACP models, interpretability analysis steps should be integrated into the model training pipeline to deeply explore the causal logic and potential relationships within peptide data. Such analysis will not only help reveal the key features affecting ACP recognition but also provide actionable insights for optimizing existing models, providing clear foundations for their clinical translation.

#### Increase the interpretability of the model

At present, the most commonly used interpretability methods in ACP models are intrinsic interpretability models and attribution‐based methods. Intrinsic interpretability models, such as decision tree, KNN, and linear regression, are interpretable because of their understandable and traceable decision‐making processes [97]. Linear and logistic regression are used to explain the relationship between independent and dependent variables through simple mathematical formulas; however, visualization or other methods are required to explain the model decisions to nonexpert users [98]. Decision tree models are interpretable because the decision‐making process is displayed in a hierarchical structure, allowing for rule extraction and intuitive understanding. This interpretability has led to the development of rule‐based deep learning models derived from decision trees [99]. KNN relies on the distance measure between samples, clearly demonstrating how the model output changes based on variations in nearby samples, but it is susceptible to the number of features and the distance function. Although these models have been applied in ACP prediction, unfortunately, they do not provide clear interpretability explanations when established. Thus, it is recommended to incorporate interpretability analysis in the development of new ACP models using these algorithms, which can directly enhance the model's transparency and credibility. In addition to these intrinsic interpretability models, RF as an ensemble model composed of multiple decision trees, also has intrinsic interpretability. By calculating feature importance, RF can reveal which features have a significant impact on the predictions of the model [100, 101], helping to understand the mechanism of the model. More importantly, RF has already been applied with interpretability in ACP prediction. In ACPred, interpretability rules are constructed based on a RF model, and AAC features are used to extract 20 key features that can distinguish ACPs from non‐ACPs based on amino acid combinations. ACPred achieved a prediction accuracy >95% with four of these features, providing an efficient and easy‐to‐understand method for predicting ACPs. Although RF offers global interpretability, the granularity of RF is relatively coarse, primarily evaluating feature importance in the overall model and preventing deep analyses of the prediction process of individual samples.

It should be noted that intrinsically interpretable models are primarily based on traditional ML models, which reveal the relationships between input features and prediction results through explicit rules and formulas. However, deep learning models perform well on prediction tasks owing to their highly nonlinear and multilayer structure, but lack an explanation framework. Attribution‐based methods were developed to address this issue. Attribution‐based methods are used to analyze the contribution of each feature to the final prediction and explain the decision‐making process of deep learning models [102, 103]. SHAP was originally proposed in the context of game theory and is a well‐established attribution‐based interpretability method (Figure 4B) [104, 105]. The SHAP method calculates all possible model permutations for each feature, ensuring that the contribution of the same feature is similar across different models, avoiding variability and instability in feature importance evaluations, and achieving feature attribution consistency [106]. SHAP is the interpretability method most commonly used for ACP models. For instance, ANNprob‐ACPs uses SHAP to identify the top 20 features in ACP prediction, where physicochemical properties alongside DPC, AAC, and PAAC are crucial to the model's performance. Quasi‐Sequence Order (QSO) functional features can reveal sequence order effects, whereas the DPC, AAC, and PAAC composition characteristics further enhance the accuracy and stability of ACP recognition [107]. Liu et al. [108] employed the SHAP Summary Plot method in ACPPfel to generate summary plots that visualized the Shapley values of each sample's features, calculated feature importance rankings and revealed the main driving factors in the model's decision‐making process. PLMACPred uses SHAP to analyze the sequence properties of the training samples, visualize the contributions of the top 25 important features, and use the MEME tool to enrich a series of ACP motifs from their sequences. Given the mature application of the SHAP method, we recommend integrating SHAP analysis into ACP deep learning models to enhance the interpretability and transparency of the models.

CAM is another attribution‐based interpretability method that helps with understanding the decision‐making process of CNNs through visualizing the specific regions in an image that strongly impact the classification result. A CAM heat map is generated by adding a global average pooling (GAP) layer added after the last convolutional layer in the model (Figure 4C). The GAP output is linearly combined to generate a class prediction, highlighting the regions associated with a specific class and the areas on which the model particularly focuses when forming predictions [109, 110, 111]. Grad‐CAM is an improved version of the CAM method that uses gradient information to weight the feature maps, generating more detailed and accurate class activation maps. Unlike CAM, Grad‐CAM does not rely on the GAP layer, making it applicable to any type of convolutional neural network and offering greater adaptability. By calculating the gradient of the target class with respect to the last convolutional layer and combining these gradients with the feature maps, Grad‐CAM reveals the specific regions the model focuses on when making decisions, making it more flexible and precise than CAM [112, 113, 114]. In ACP prediction, Grad‐CAM is commonly used to analyze the interpretability of ACP convolutional models. xDeep‐AcPEP, a model trained using a CNN framework, uses Grad‐CAM for interpretability analysis. Contribution factor analysis showed that the N‐ and C‐termini of the ACP had the greatest impact on the model's prediction, indicating that the terminal regions were closely related to the activity. This effect may be attributed to specific structural or physicochemical properties that enhance a peptide's affinity for cancer cell membranes, thereby improving its penetration ability and exerting anticancer activity. Additionally, certain terminal residues, such as M, S, A, T, V, and G, increase the stability of ACPs in vivo [115]. Given that Grad‐CAM is particularly suitable for various CNNs and their variants, we recommend integrating the Grad‐CAM method when developing new ACP models using CNNs to enhance the model's interpretability.

#### Visualization of interpretability methods

In explainable artificial intelligence, visualization is one of the key techniques for enhancing model transparency and interpretability. The introduction of visualization techniques in ACP models allows users to intuitively understand the model's decision‐making process, revealing how the model responds to input data and which features or key areas play a critical role in the prediction [116, 117]. Common visualization methods include feature importance plots and heat maps.

Feature importance plots depict the impact of each feature on the final prediction result. These plots are typically generated using tree‐based models that assess feature importance through calculating their contributions to decision tree splits. The THPep model, which predicts tumor‐targeted peptides, and the PAAP model, which predicts antihypertensive peptides, use RF features to directly generate feature importance rankings [118, 119]. In addition, SHAP interpretability methods can be used to directly generate feature importance plots to display the contributions of each feature to the prediction. The SHAP feature importance plots include the SHAP summary, dependence, and force plot [120]. The SHAP summary plot shows the contribution and influence of each feature to the model output and its influence on feature values. The SHAP dependence plot depicts the relationship between the features and their SHAP values, whereas the SHAP force plot helps with understanding the specific contribution of each feature to individual sample predictions [121, 122]. The ANNprob‐ACP model uses a SHAP summary plot to display the top 20 features that most strongly contribute to detecting ACPs. The SHAP summary plot shows the key roles played by physicochemical properties and compositional characteristics in model performance. Additionally, SHAP was combined with t‐SNE dimensionality‐reduction techniques. t‐SNE is a dimensionality reduction method that is used to map high‐dimensional data into a two‐dimensional space to help with understanding and visualizing the similarity and clustering of high‐dimensional features. The pACP‐HybDeep model uses SHAP‐ and t‐SNE‐based feature analysis methods for visualizing the results and the effectiveness of the extracted features [123].

Heatmaps are also a common visualization tool used for model interpretability, helping to display the importance distribution of model input features in space or time. In addition to the CAM mentioned earlier, attention mechanisms can also generate attention heatmaps. Attention mechanisms dynamically adjust the focus on each feature through calculating the correlation between the input features and the current target. The model generates attention heat maps through assigning a set of attention weights to each input feature, reflecting the degree of importance placed by the model on each feature during the decision‐making process [124, 125]. In the interpretability analysis of ACP models, attention heatmaps have not been widely adopted as a visualization method, but they have been preliminarily applied in the interpretability analysis of other prediction models. Zhang et al. [126] used attention mechanisms in Umami‐BERT to calculate the attention values for each amino acid and generated attention heat maps, revealing the level of focus of the model on different amino acids when processing umami peptides. Shuai et al. [127] developed the ConTCR model, which uses attention‐score heat maps for visualizing the results and captures information regarding TCR‐pMHC interactions. This model provides an understanding of key TCR‐pMHC interactions by combining the sequence features of pMHC and TCR through a contrastive learning strategy and strengthening the focus on critical interactions via attention mechanisms. In the future, with the widespread application of attention mechanisms in ACP models, attention heatmaps are expected to become an important tool for ACP interpretability visualization, helping to enhance the transparency of the model.

### Wet‐laboratory experiments

#### Absence of wet‐laboratory experiments

A key step in AI use in science is integrating AI with biological wet‐laboratory experiments to increase research efficiency and overcome the limitations of traditional computational models. The University of Washington determined the structure of the nuclear pore complex using an advanced AlphaFold model and cryoelectron microscopy. AlphaFold efficiently predicts structures; however, its accuracy is limited by the training data: the results may be biased. This shows that computational models cannot completely replace biological experiments [128]. Shan et al. [129] developed an AI model for optimizing antibodies using geometric deep learning methods, combining a small number of wet‐laboratory experiments to achieve extensive neutralizing activity optimization for SARS‐CoV‐2 variants. Due to the numerous mutation sites of the COVID‐19 virus and limited prior information, expert experience was constrained. They adopted the “AI model + a small number of wet‐laboratory experiments” approach, using the AI model to expand the scientific hypothesis space, thereby enhancing the antibody's affinity and efficacy. Unfortunately, although many models in ACP have achieved good performance at the theoretical and computational levels, only a few models have been validated through biological experiments. Out of the 68 ACP models, only 3 models included biological experiment validation during their development. This lack of validation directly affects the model ability to be translated into clinical applications. The absence of wet‐laboratory validation prevents a full assessment of the model generalization ability, which is crucial for clinical applications. Laboratory results often fail to fully reflect the complexity of clinical practice, as factors such as patient variations, environmental influences, and drug sensitivity can affect the actual efficacy of ACPs. Models that have not been validated through wet‐laboratory experiments may not accurately predict the biological activity, stability, and safety of ACPs in different clinical settings, thus reducing their effectiveness and application value in clinical treatments [130, 131]. Thereby, the combination of ACP models and biological wet‐laboratory experiments is a crucial step in evaluating the actual effectiveness of ACP models and ensuring their successful translation into clinical treatment options.

#### ACP wet‐laboratory methods

We summarized and found that out of the 68 models, 3 models were validated through wet‐laboratory experiments from both in vivo and in vitro perspectives. Kao et al. [132] validated the ACPs selected by an AI model. The peptides were dissolved and diluted to appropriate concentrations, and their anticancer activity was assessed using the MTT assay. The IC50 value was determined using a dose–response curve, confirming the anticancer activity and selective cytotoxicity of the peptides. Xu et al. [133] developed a classification model for ACPs by integrating deep learning and ML, and validated the anticancer effects of two predicted ACPs through in vitro cell experiments. In the in vitro experiments, the SRB method was used to assess the cytotoxicity of the peptides on 4T1 mouse breast cancer cells, and IC50 values were calculated, further validating the accuracy of the model's predictions. CNBT‐ACPred underwent comprehensive wet‐laboratory validation during its development, encompassing both in vivo and in vitro evaluations. In the in vitro experiments, various concentrations of the candidate ACPs were tested on B16F10 and K562 cancer cells to evaluate their anticancer activity and selectivity. The effects of the peptides on the cells were comprehensively validated using the SRB method, LDH release assay, Calcein‐AM/PI dual staining, and transmission electron microscopy (TEM). In the in vivo experiments, the anticancer effects of the peptides were further evaluated using a mouse xenograft tumor model. In these experiments, tPep14 demonstrated significant anticancer effects with high selective cytotoxicity, validating the accuracy and effectiveness of the model's predictions. These experimental results support the practical application potential of the CNBT‐ACPred model in ACP screening [134].

In conclusion, we recommend verifying the ACPs identified with AI‐based models through in vitro and in vivo experiments. In vitro experiments should be used to assess the cytotoxicity and selectivity of ACPs using various cancer cell lines using common methods, such as the MTT assay, SRB method, LDH release assay, and dual calcein‐AM/PI staining, to validate ACP activity and mechanism [135, 136, 137, 138]. Similarly, in vivo xenograft tumor models should be used to evaluate the anticancer effects of ACPs, such as tumor growth, volume changes, and histological analyses, to further validate the real‐world efficacy of ACPs [139, 140]. After sufficient animal experimentation and validation, the ACP can proceed to the clinical trial phase to assess the safety, efficacy, and dosage of the new ACP, ultimately leading to its clinical translation. This dry‐wet closed‐loop model provides a feedback mechanism, where AI screens for ACPs through large‐scale data mining, and wet experiments are conducted to validate their anticancer effects and selective toxicity. The validated ACPs provide new training datasets that can be used for further optimizing the AI model. The dry‐wet closed‐loop method allows AI models to continuously learn and iterate, driving the discovery and clinical translation of new ACPs.

## 4 INTEGRATION OF ACP MODELS WITH OTHER TECHNOLOGIES

**Figure 5:**
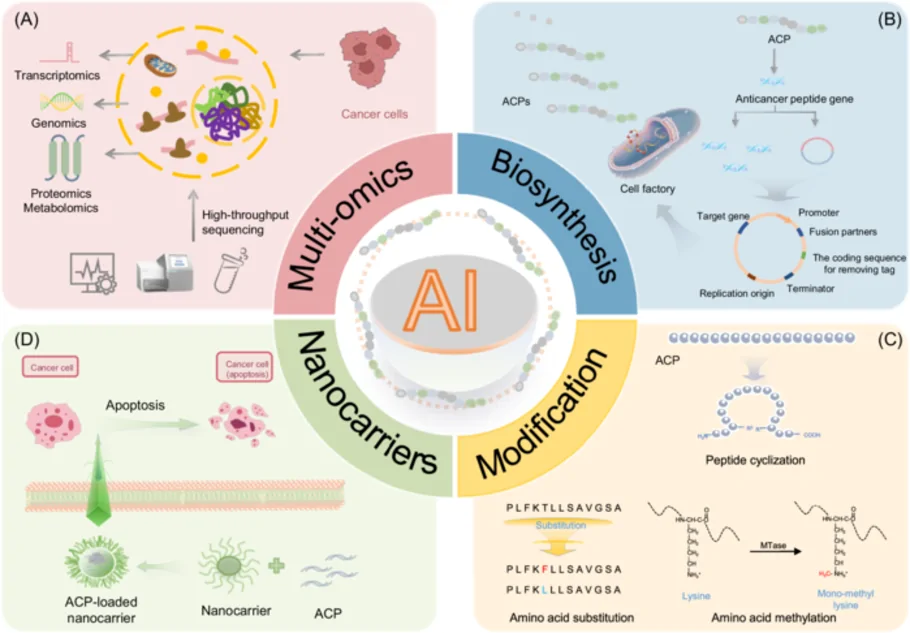
Integrating AI‐selected peptides with multi‐omics technologies and other biotechnologies. (A) Multi‐omics approach combining transcriptomics, genomics, proteomics, and metabolomics for anticancer peptide (ACP) identification. (B) Biosynthesis of ACPs using cell factories for peptide production. (C) ACP modification through peptide cyclization, amino acid substitutions, or methylation to enhance stability. (D) ACP‐loaded nanocarriers for targeted cancer therapy, improving treatment efficacy.

Although AI has made progress in ACP screening, there are still many challenges in achieving the actual development of anticancer drugs from sequences. The development of peptide‐based drugs is often limited by high costs, difficulties in large‐scale production, and susceptibility to enzymatic degradation. Furthermore, the current size of peptide candidate libraries is limited, and there is a lack of relevant information in public databases, which poses a significant constraint on efficient ACP design and limits the widespread application of AI models. Thus, ACPs identified through AI screening still need to be integrated with technologies such as multi‐omics, synthetic biology, amino acid modification techniques, and nanodelivery systems to expand the ACP database, reduce peptide production costs, and enhance the clinical application of peptides, ultimately leading to the development of new, safe, and effective anticancer drugs (Figure 5A‒D).

### Multi‐omics

The application of multi‐omics in the screening of other bioactive peptides has been widely explored, but the integration of AI with multi‐omics for systematic screening in ACP research is still in its early stages. By integrating multi‐level omics data, such as differential genes, differential proteins, and differential metabolites, a more comprehensive peptide candidate library can be provided for AI models. This multi‐dimensional data integration helps to identify key biomarkers associated with cancer pathology and also aids in the discovery of potential ACP sequences [141]. Multi‐omics can precisely locate important gene expression changes in cancer cells, as well as the differences in proteins and metabolites closely related to tumor initiation and progression. For example, genomics can reveal gene mutations and gene expression changes in tumor cells, with potential peptide information provided through small open reading frame (sORF) prediction. Transcriptomics data can reveal gene expression differences in tumor tissues, while proteomics directly reflects functional information of tumor‐related proteins, further helping to screen and optimize ACPs [142]. Metabolomics data, on the other hand, reveal changes in metabolic pathways and differences in metabolic products in tumor cells (Figure 5A) [143]. By combining these omics data, AI models can screen potential peptide molecules based on the expression patterns of differential genes and proteins, and discover potential ACP candidates from metabolomics data, further predicting their efficacy and stability. Ma et al. [144] applied human gut microbiome and metaproteomics data to construct a library containing 2349 antimicrobial peptide candidates, which were screened using deep learning models. Ultimately, 11 peptides that showed strong antibacterial effects against drug‐resistant Gram‐negative bacteria were selected. In vivo experiments showed that these peptides significantly reduced bacterial load in a mouse lung bacterial infection model, demonstrating good therapeutic potential. In the future, integrating comparative analyses of gut microbiome profiles between healthy individuals and cancer patients, with multi‐metabolomics data, may enable the identification of specific microbiomes and their differential peptide datasets that are closely linked to cancer occurrence and progression. AI models can screen for potential ACPs related to changes in the gut microbiome based on these differential datasets.

Multi‐omics approaches provide a vast resource of ACP candidate libraries and enrich the natural ACP database, facilitating the elucidation of ACP mechanisms of action and offering reference templates for the efficient design of ACPs. Currently, public ACP databases mainly include sequence information, physicochemical properties, and other data, but the analysis of their mechanisms of action is still insufficient. These databases lack a deep understanding of ACPs' interactions with cancer cell‐related targets, immune system responses, and metabolic pathways. However, the integration of multi‐omics data can address this gap. Through multi‐omics sequencing, the specific action sites and targets of ACPs can be revealed [145]. Among these, spatial omics stands out by capturing and analyzing the spatial distribution of molecules and cells within tissues, and can provide spatial dimension data on the interactions between anticancer drugs and their targets. This offers a new perspective on the action of anticancer drugs in specific cellular and tumor microenvironments, revealing the targeted anticancer mechanisms of drug molecules in different regions [146]. Moreover, as ACP data continues to grow, multi‐omics data provides an ideal training set for new regression models and supports the construction of generative models. Regression models, by learning the relationships between ACP sequences, structures, and functions, can accurately predict the anticancer activity of peptides and optimize their structural design. Generative models can generate new anticancer candidates based on existing multi‐omics data, further expanding the ACP library. Combined with explainable AI technology, these models can deeply analyze the structure‐function relationships of ACPs. The integration of AI enables the successful design of ACPs with enhanced target specificity, high affinity, and biological stability, thereby enhancing their efficacy in cancer treatment and advancing the development of precision drugs.

### Synthetic biology

The synthesis of ACPs and other bioactive peptides selected by AI mainly relies on chemical synthesis methods. Chemical synthesis is costly, and due to its complexity, it is difficult to ensure that the peptide's structure meets the expected specifications, which may result in structural deviations. For instance, it is difficult to achieve the stability of disulfide bonds in peptide chains during chemical synthesis, leading to incomplete folding of the peptide and reduced binding ability with target proteins. The presence of cysteine residues may result in intermolecular disulfide bonds or impurities from multimolecular aggregation. As the number of thiol groups that need to be paired increases, disulfide bonds within the peptide chain may mispair or fail to pair, significantly increasing the complexity of the structure [147, 148]. Additionally, the reagents used in the chemical synthesis process may have negative environmental impacts, limiting their sustainability and economic feasibility for large‐scale production. To address these issues, synthetic biology technologies, through gene editing and the application of cell factories, provide new solutions [149]. Using microorganisms or engineered cell factories for large‐scale production of ACPs reduces production costs and avoids the byproduct issues associated with chemical synthesis (Figure 5B). With the advancement of synthetic biology technologies, some research teams have begun to introduce the peptide's gene sequences into expression vectors, utilizing host systems to achieve the biosynthesis of peptides. Pei et al. [150] utilized engineered synthetic biology technology to successfully heterologously express Mechercharmycin A in *Escherichia coli*, which has significant anticancer activity and a unique structure. Pasupuleti et al. [151] investigated a synthetic retro dipeptide, R‐DIM‐P‐LF11‐215, derived from lactoferricin, and heterologously expressed it in *Escherichia coli*. After purification by affinity chromatography, they found that this fusion protein could selectively target and kill Gb3+ cancer cells.

### Amino acid modification technologies

To enhance the therapeutic effect and clinical application potential of ACPs, peptide sequences selected by AI may require a series of modifications and optimizations. Bioactive peptides are often sensitive to proteases and can easily lose their activity when enzymatically degraded in vivo. Consequently, amino acid modifications or substitutions have emerged as a new strategy to enhance the stability and bioactivity of peptides (Figure 5C). For example, cyclization can enhance the structural stability of peptides, reduce their sensitivity to enzymatic degradation, and increase their bioactivity and targeting ability. Cyclic peptides typically exhibit stronger antimicrobial and anticancer activities than linear peptides, making cyclization a key method for optimizing ACP performance. Phakellistatin 13 is a cyclic heptapeptide composed of alternating l‐ and d‐amino acid residues exhibits potent anti‐hepatocellular carcinoma activity by inducing cell cycle arrest by modulating the p53 and MAPK signaling pathways [152]. Another effective modification method can effectively enhance anticancer activity by substituting or adding specific amino acids. Hadianamrei et al. [153] enhanced the selectivity of ACPs and reduced toxicity to normal cells by replacing lysine (K) with arginine (R), while adding two isoleucine (I) residues at the C‐terminus increased the peptide's hydrophobicity and α‐helix content, thereby improving its anticancer activity. In addition to cyclization and amino acid substitution, methylation of substrates can also effectively regulate the function and stability of peptides. Methylation, as an important epigenetic modification, can significantly affect the binding ability of peptides to their targets and their biological activity. Methylation alters the structure and function of RNA molecules by adding methyl groups. In this process, methylation regulates the epigenetic state of RNA, enhancing the specificity and stability of peptides towards their targets and improving the anticancer effects of the peptides. Mtp1 is a peptide composed of 12 amino acids that specifically binds to m(6)A‐modified RNA. This peptide enhances anticancer effects by inhibiting the binding of the FTO protein, preventing m(6)A demethylation, and increasing the retention level of m(6)A modification [154]. Methylation can not only alter RNA stability and translation efficiency but also influence RNA secondary structure and interactions with proteins. In summary, methylation provides new ideas and possibilities for the modification of ACPs.

### Nanodelivery systems

In addition to being susceptible to protease degradation, ACPs selected by AI may also be constrained by multiple factors, such as cellular phagocytosis and organ excretion, making it difficult for them to effectively cross the cell membrane and release their therapeutic effects within target cells. Furthermore, factors such as hypoxia, acidic conditions, and high concentrations of reactive oxygen species in the tumor microenvironment can lead to the degradation of chemical structures like sulfur groups and amide bonds in ACPs, affecting their stability. The advent of nanotechnology offers new approaches to overcome these challenges [155]. Through strategies like cell‐specific targeting and molecular transport, nanocarriers can effectively overcome bio‐distribution and intracellular transport barriers found in traditional drug delivery systems, regulate the blood circulation time of ACPs, thus increasing their concentration at tumor sites, enhancing the therapeutic effect. Using biocompatible materials derived from natural sources enhances the safety of ACPs by minimizing potential toxicity and improving their compatibility for clinical applications (Figure 5D) [156]. DermaseptinPP is an ACP with broad antitumor activity, but high doses may trigger severe hemolytic reactions. To address the biosafety issue of DermaseptinPP, pH‐sensitive membrane materials were used to load it into nanoliposomes, creating a system with pH‐responsive properties. The lipophilicity of the liposome surface was reduced, thereby minimizing hemolytic toxicity. When the environmental pH falls below 6.5, the chemical bonds in the liposomes break, releasing Dermaseptin‐PP and effectively killing tumor cells [157]. FA‐EEYSV‐NH₂ is a novel anticancer prodrug that combines a folic acid (FA) targeting recognition site, a dipeptide linker, and ACPs. This peptide molecule can self‐assemble into nanoparticles in a pH of 7.0, while forming nanofibers in weak acidic conditions. This self‐assembly property enhances the stability of ACPs and promotes their targeted delivery and release within the tumor microenvironment [158].

## 5 AI‐DRIVEN ACP SCREENING FRAMEWORK

**Figure 6:**
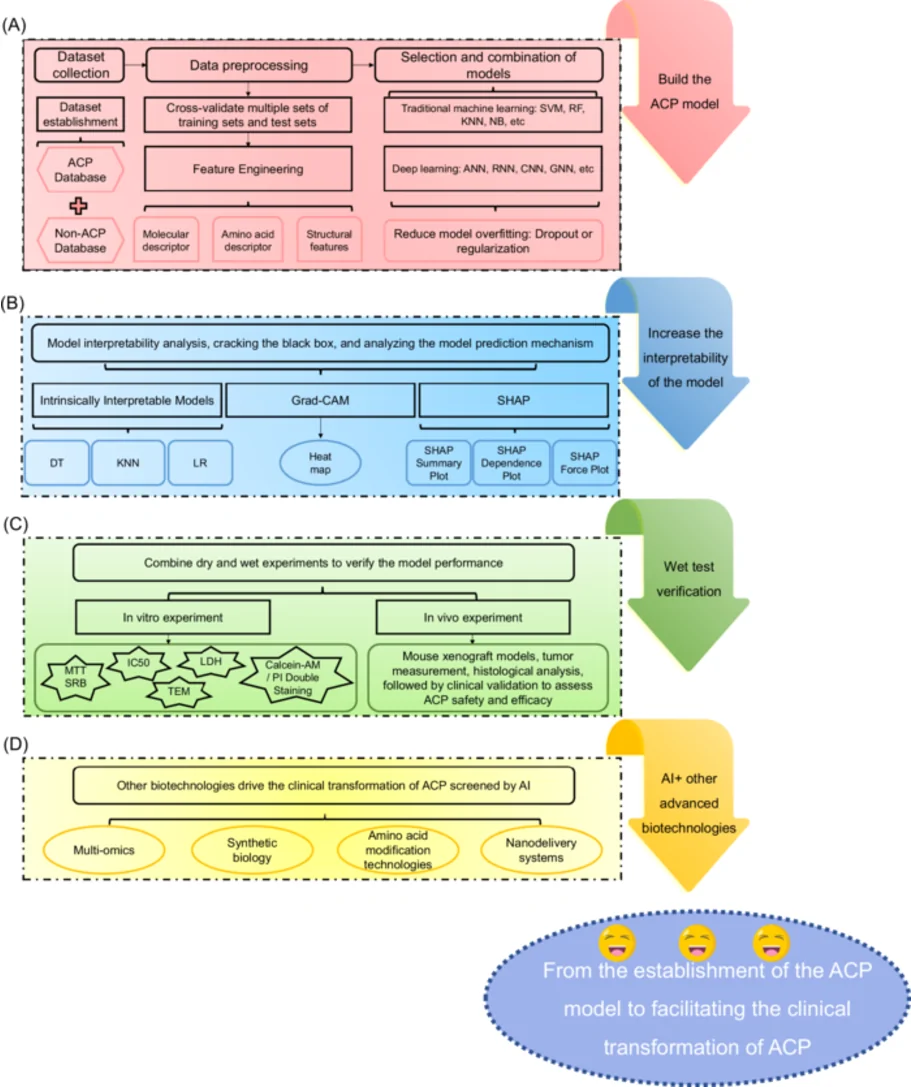
Comprehensive AI‐based anticancer peptide (ACP) screening framework. (A) Data set collection, preprocessing, and model training. (B) Model interpretability analysis using methods like Grad‐CAM and SHAP. (C) Wet‐laboratory validation of ACP through in vitro and in vivo experiments. (D) Other biotechnologies drive the clinical transformation of ACPs screened by AI.

We summarized a comprehensive AI‐driven ACP screening framework based on the review of prior models (Figure 6A‒D). This framework is centered on combining AI with interpretability analysis and wet‐laboratory validation, integrating various biotechnologies to enable the rapid screening and efficient application of ACPs. The framework comprises several key steps: collecting and organizing datasets, extracting features, training and optimizing the model, analyzing model interpretability, conducting wet‐laboratory validation, and integrating ACPs with other biotechnologies. The first step in the framework involves comprehensively collecting ACP datasets using CD‐HIT or other data augmentation techniques to ensure the consistency of the positive and negative datasets and constructing a unified test set to ensure fairness and comprehensiveness while avoiding data leakage. Once the datasets are prepared, feature extraction techniques, including molecular descriptors, amino acid descriptors, and structural features are applied to extract relevant features from the peptide sequences. Traditional ML or modern deep learning methods, combined with cross‐validation, are then used to train and optimize the model. To mitigate overfitting, regularization techniques or dropout layers are introduced (Figure 6A). Second, explainable AI is thus incorporated into ACP model development via applying interpretability methods such as SHAP and Grad‐CAM, and using visualization tools to interpret the prediction mechanisms of the model. This process avoids the black‐box issue and increases the interpretability and credibility of the model (Figure 6B). Third, the model is tested through in vitro and in vivo wet‐laboratory experiments to validate the effects of the identified ACPs. In vitro testing focuses on assessing the anticancer effects of the ACPs on cancer cells, including their ability to inhibit cell proliferation, induce apoptosis, and evaluate toxicity through hemolysis and cytotoxicity assays. In vivo, mouse models are used to assess ACP efficacy in reducing tumor growth, preventing metastasis, and evaluating overall survival. Toxicity studies in mice monitor organ damage, immune response, and potential side effects, providing essential data for their clinical application (Figure 6C). Finally, the framework incorporates AI with multi‐omics, synthetic biology, amino acid modification, and nanodelivery technologies to overcome the limitations of ACPs for the development of new ACP drugs (Figure 6D). This framework involves a progressive and integrated process, in which the results of the validation in each stage can be fed back into subsequent stages. The wet‐laboratory validation results and sequence modification outcomes provide new data for the model, enriching the data set and promoting iterative updates to the model, continuously increasing predictive ability.

## 6 CONCLUSION

To outline a complete AI‐based ACP screening framework, we detailed the summary of 68 ACP models. Based on the training processes and limitations of these 68 models, we presented a comprehensive ACP screening framework that includes data set collection and organization, feature extraction, model training and optimization, interpretability analysis, and wet‐laboratory validation. Additionally, this framework integrates multi‐omics, synthetic biology, amino acid modification technologies, and nanocarrier technologies to facilitate the process from model development to clinical application. These technologies should be integrated to increase the biological activity, structural stability, and manufacturability of AI‐selected ACPs. In conclusion, AI‐driven ACP discovery holds great potential. It can expedite peptide screening and functional prediction, significantly enhancing the efficiency and accuracy of novel peptide‐based anticancer drug development, thereby offering more diverse and effective treatment options for cancer.

## AUTHOR CONTRIBUTIONS

**Junrui Wu**: Writing—original draft; funding acquisition; investigation; conceptualization; validation; visualization; project administration; resources; supervision; data curation; writing—review and editing. **Shuaiqi Ji**: Writing—original draft; writing—review and editing; conceptualization; investigation; methodology; validation; software; formal analysis; data curation; visualization. **Kashif Iqbal Sahibzada**: Writing—original draft; conceptualization; validation; methodology; formal analysis; supervision; data curation. **Mengxue Lou**: Investigation; conceptualization; methodology; validation; formal analysis; software; data curation. **Feiyu An**: Investigation; validation; formal analysis; data curation; supervision; conceptualization. **Wenqian Li**: Conceptualization; validation; methodology; software; formal analysis; data curation. **Jiawei Guo**: Investigation; validation; formal analysis; data curation; visualization; methodology. **Taowei Zhang**: Software; formal analysis; data curation; supervision; methodology. **Xinyi Zhang**: Conceptualization; investigation; formal analysis; data curation. **Yilin Chou**: Data curation; software; methodology; conceptualization. **Henan Zhang**: Investigation; validation; formal analysis; data curation. **Hao Jin**: Validation; visualization; formal analysis. **Teng Ma**: Investigation; methodology; software. **Weichi Liu**: Conceptualization; methodology; data curation. **Begali Alikulov**: Validation; visualization; investigation. **Natalia Alekseevna Golovneva**: Investigation; data curation; conceptualization. **Hooi Ling Foo**: Conceptualization; methodology. **Issayeva Kuralay**: Validation; formal analysis. **Zhihong Sun**: Writing—original draft; writing—review and editing; project administration; supervision; visualization; data curation; resources. **Dongqing Wei**: Writing—original draft; writing—review and editing; supervision; data curation; resources; project administration. **Rina Wu**: Funding acquisition; writing—original draft; writing—review and editing; visualization; project administration; resources; supervision; data curation; formal analysis; software; conceptualization. All authors have read the final manuscript and approved it for publication.

## CONFLICT OF INTEREST STATEMENT

The authors declare no conflicts of interest.

## ETHICS STATEMENT

No animals or humans were involved in this study.

## Supporting information

Supporting Information.

**Table S1.** Summary of 68 ACP prediction models.
**Table S2.** A summary of public databases for ACPs.
**Table S3.** Amino acid descriptor methods for feature extraction of ACP sequences in AI models.

## Data Availability

This manuscript does not generate any new script or data. Supplementary materials (graphical abstract, slides, videos, Chinese translated version, and update materials) may be found in the online DOI or iMeta Science http://www.imeta.science/imetaomics/.
