# Supplementary material for: Artificial intelligence‐driven anticancer peptide discovery
Source: IMetaOmics. 2025 Nov 5;2(4):e70063. doi: 10.1002/imo2.70063 (PMC12806129; doi:10.1002/imo2.70063)
Supplement: Supplementary file 1 — Supporting Information. [file IMO2-2-e70063-s002.docx]

Supporting information to

**Artificial Intelligence-Driven Anticancer Peptide Discovery**

**Running title**: **AI-driven ACP discovery**

Junrui Wu^1#^, Shuaiqi Ji^1#^, Kashif Iqbal Sahibzada^2,3,4#^, Mengxue Lou^1^, Feiyu An^1^, Wenqian Li^1^, Jiawei Guo^1^, Taowei Zhang^1^, Xinyi Zhang^1^, Yilin Chou^1^, Henan Zhang^1^, Hao Jin^5^, Teng Ma^5^, Weichi Liu^5^, Begali Alikulov^6^, Natalia Alekseevna Golovneva^7^, Hooi Ling Foo^8,9^, Issayeva Kuralay^10^, Zhihong Sun^5^*, Dongqing Wei^4^*, Rina Wu^1^*

^1^College of Food Science, Shenyang Agricultural University, National Agricultural Environmental Microbial Germplasm Resource Bank, Liaoning Engineering Research Center of Food Fermentation Technology, Shenyang Key Laboratory of Microbial Fermentation Technology Innovation, Shenyang 110866, PR China

^2^College of Biological Engineering, Henan University of Technology, Zhengzhou 450001, PR China

^3^Department of Health Professional Technologies, Faculty of Allied Health Sciences, The University of Lahore, Lahore 54570, Pakistan

^4^State Key Laboratory of Microbial Metabolism, Joint International Research Laboratory of Metabolic and Developmental Sciences, School of Life Sciences and Biotechnology, Shanghai Jiao Tong University, 800 Dongchuan Road, Shanghai 200240, PR China

^5^Key Laboratory of Dairy Biotechnology and Engineering, Ministry of Education, Inner Mongolia Agricultural University, Hohhot 010018, PR China

^6^Department of Biotechnology, Samarkand State University, Samarkand, 140104, Uzbekistan

^7^Institute of Microbiology of the National Academy of Sciences of Belarus, Minsk, ul. Kuprevicha, 2, 220141, Belarus

^8^Department of Bioprocess Technology, Faculty of Biotechnology and Biomolecular Sciences, Universiti Putra Malaysia, 43400 UPM Serdang, Selangor, Malaysia

^9^Lactic Acid Bacteria Biota Technology Research Program, Research Laboratory of Probiotics and Cancer Therapeutics, UPM-MAKNA Cancer Research Laboratory (CANRES), Institute of Bioscience, Universiti Putra Malaysia, 43400 UPM Serdang, Selangor, Malaysia

^10^Department of Biotechnology, Toraighyrov University, Pavlodar, 140008, Kazakhstan

^#^These authors contributed equally: Junrui Wu, Shuaiqi Ji, Kashif Iqbal Sahibzada

*Correspondence:

wrn6956@163.com (Rina Wu), dqwei@sjtu.edu.cn (Dongqing Wei), sunzhihong78@163.com (Zhihong Sun)

**SUPPORTING TABLES**

**Table S1** Summary of 68 ACP prediction models.

**Table S2** A summary of public databases for ACPs.

**Table S3** Amino acid descriptor methods for feature extraction of ACP sequences in AI models.

**REFERENCES**

1. Salam, Abdu, Faizan Ullah, Farhan Amin, Izaz Ahmad Khan, Eduardo Garcia Villena, Angel Kuc Castilla, Isabel de la Torre. 2024. “Efficient prediction of anticancer peptides through deep learning.” *Peerj Computer Science* 10: e2171. <https://doi.org/10.7717/peerj-cs.2171>

2. Azad, Humera, Muhammad Yasir Akbar, Jawad Sarfraz, Waseem Haider, Muhammad Naeem Riaz, Ghulam Muhammad Ali, Shakira Ghazanfar. 2024. “G-ACP: a machine learning approach to the prediction of therapeutic peptides for gastric cancer.” *Journal of Biomolecular Structure & Dynamics* 1-14. <https://doi.org/10.1080/07391102.2024.2323141>

3. Ullah, Faizan, Abdu Salam, Muhammad Nadeem, Farhan Amin, Hussain AlSalman, Mohammad Abrar, Taha Alfakih. 2024. “Extended dipeptide composition framework for accurate identification of anticancer peptides.” *Scientific Reports* 14: 17381. <https://doi.org/10.1038/s41598-024-68475-8>

4. Zhang, Lichao, Xueli Hu, Kang Xiao, Liang Kong. 2024. “Effective identification and differential analysis of anticancer peptides.” *Biosystems* 241: 105246. <https://doi.org/10.1016/j.biosystems.2024.105246>

5. Zhong, Guolun, Lei Deng. 2024. “ACPScanner: prediction of anticancer peptides by integrated machine learning methodologies.” *Journal of Chemical Information and Modeling* 64: 1092-1104. <https://doi.org/10.1021/acs.jcim.3c01860>

6. Kao, Hui-Ju, Tzu-Han Weng, Chia-Hung Chen, Yu-Chi Chen, Yu-Hsiang Chi, Kai-Yao Huang, Shun-Long Weng. 2024. “Integrating in silico and in vitro approaches to identify natural peptides with selective cytotoxicity against cancer cells.” *International Journal of Molecular Sciences* 25: 6848. <https://doi.org/10.3390/ijms25136848>

7. Sangaraju, Vinoth Kumar, Nhat Truong Pham, Leyi Wei, Xue Yu, Balachandran Manavalan. 2024. “mACPpred 2.0: Stacked deep learning for anticancer peptide prediction with integrated spatial and probabilistic feature representations.” *Journal of Molecular Biology* 436: 168687. <https://doi.org/10.1016/j.jmb.2024.168687>

8. Xu, Meiqi, Jiefu Pang, Yangyang Ye, Ziyi Zhang. 2024. “Integrating traditional machine learning and deep learning for precision screening of anticancer peptides: A novel approach for efficient drug discovery.” *Acs Omega* 9: 16820-16831. <https://doi.org/10.1021/acsomega.4c01374>

9. Danish, Sufyan, Asfandyar Khan, L. Minh Dang, Mohammed Alonazi, Sultan Alanazi, Hyoung-Kyu Song, Hyeonjoon Moon. 2024. “Metaverse applications in bioinformatics: A machine learning framework for the discrimination of anti-cancer peptides.” *Information* 15: 48. <https://www.mdpi.com/2078-2489/15/1/48>

10. Liang, Xiao, Haochen Zhao, Jianxin Wang. 2024. “MA-PEP: A novel anticancer peptide prediction framework with multimodal feature fusion based on attention mechanism.” *Protein Science* 33: e4966. <https://doi.org/10.1002/pro.4966>

11. Bian, Jilong, Xuan Liu, Guanghui Dong, Chang Hou, Shan Huang, Dandan Zhang. 2024. “ACP-ML: A sequence-based method for anticancer peptide prediction.” *Computers in Biology and Medicine* 170: 108063. <https://doi.org/10.1016/j.compbiomed.2024.108063>

12. Karim, Tasmin, Md. Shazzad Hossain Shaon, Md. Fahim Sultan, Md. Zahid Hasan, Kafy Abdulla - Al. 2024. “ANNprob-ACPs: A novel anticancer peptide identifier based on probabilistic feature fusion approach.” *Computers in Biology and Medicine* 169: 107915. <https://doi.org/10.1016/j.compbiomed.2023.107915>

13. Arif, Muhammad, Saleh Musleh, Huma Fida, Tanvir Alam. 2024. “PLMACPred prediction of anticancer peptides based on protein language model and wavelet denoising transformation.” *Scientific Reports* 14: 16992. <https://doi.org/10.1038/s41598-024-67433-8>

14. Zhang, Shengli, Ya Zhao, Yunyun Liang. 2024. “AACFlow: an end-to-end model based on attention augmented convolutional neural network and flow-attention mechanism for identification of anticancer peptides.” *Bioinformatics* 40: btae142. <https://doi.org/10.1093/bioinformatics/btae142>

15. Niu, Yupeng, Zhenghao Li, Ziao Chen, Wenyuan Huang, Jingxuan Tan, Fa Tian, Tao Yang, Yamin Fan, Jiangshu Wei, Jiong Mu. 2024. “Efficient screening of pharmacological broad-spectrum anti-cancer peptides utilizing advanced bidirectional Encoder representation from Transformers strategy.” *Heliyon* 10: e30373. <https://doi.org/10.1016/j.heliyon.2024.e30373>

16. Yuan, Qitong, Keyi Chen, Yimin Yu, Nguyen Quoc Khanh Le, Matthew Chin Heng Chua. 2023. “Prediction of anticancer peptides based on an ensemble model of deep learning and machine learning using ordinal positional encoding.” *Briefings in Bioinformatics* 24: bbac630. <https://doi.org/10.1093/bib/bbac630>

17. Garai, Swarnava, Juanit Thomas, Palash Dey, Deeplina Das. 2024. “LGBM-ACp: an ensemble model for anticancer peptide prediction and in silico screening with potential drug targets.” *Molecular Diversity* 28: 1965-1981. <https://doi.org/10.1007/s11030-023-10602-0>

18. Li, Yanjuan, Di Ma, Dong Chen, Yu Chen. 2023. “ACP-GBDT: An improved anticancer peptide identification method with gradient boosting decision tree.” *Frontiers in Genetics* 14: 1165765. <https://doi.org/10.3389/fgene.2023.1165765>

19. Deng, Yiting, Shuhan Ma, Jiayu Li, Bowen Zheng, Zhibin Lv. 2023. “Using the random forest for identifying key physicochemical properties of amino acids to discriminate anticancer and Non-anticancer peptides.” *International Journal of Molecular Sciences* 24: 10854. <https://doi.org/10.3390/ijms241310854>

20. Yao, Lantian, Wenshuo Li, Yuntian Zhang, Junyang Deng, Yuxuan Pang, Yixian Huang, Chia-Ru Chung, Jinhan Yu, Ying-Chih Chiang, Tzong-Yi Lee. 2023. “Accelerating the discovery of anticancer peptides through deep forest architecture with deep graphical representation.” *International Journal of Molecular Sciences* 24: 4328. <https://doi.org/10.3390/ijms24054328>

21. Deng, Hua, Meng Ding, Yimeng Wang, Weihua Li, Guixia Liu, Yun Tang. 2023. “ACP-MLC: A two-level prediction engine for identification of anticancer peptides and multi-label classification of their functional types.” *Computers in Biology and Medicine* 158: 106844. <https://doi.org/10.1016/j.compbiomed.2023.106844>

22. Yang, Xuetong, Junru Jin, Ruheng Wang, Zhongshen Li, Yu Wang, Leyi Wei. 2023. “CACPP: A contrastive learning-based siamese network to identify anticancer peptides based on sequence only.” *Journal of Chemical Information and Modeling* 64: 2807-2816. <https://doi.org/10.1021/acs.jcim.3c00297>

23. Abbas, Ayad Rodhan, Bashar Saadoon Mahdi, Osamah Younus Fadhil. 2022. “Breast and lung anticancer peptides classification using N-grams and ensemble learning techniques.” *Big Data and Cognitive Computing* 6: 40. <https://www.mdpi.com/2504-2289/6/2/40>

24. Phan, Le Thi, Hyun Woo Park, Thejkiran Pitti, Thirumurthy Madhavan, Young-Jun Jeon, Balachandran Manavalan. 2022. “MLACP 2.0: An updated machine learning tool for anticancer peptide prediction.” *Computational and Structural Biotechnology Journal* 20: 4473-4480. <https://doi.org/10.1016/j.csbj.2022.07.043>

25. Liu, Jingjing, Minghao Li, Xin Chen. 2022. “AntiMF: A deep learning framework for predicting anticancer peptides based on multi-view feature extraction.” *Methods* 207: 38-43. <https://doi.org/10.1016/j.ymeth.2022.07.017>

26. Alsanea, Majed, Abdulsalam S. Dukyil, Afnan, Bushra Riaz, Farhan Alebeisat, Muhammad Islam, Shabana Habib. 2022. “To assist oncologists: An efficient machine learning-based approach for anti-cancer peptides classification.” *Sensors* 22: 4005. <https://doi.org/10.3390/s22114005>

27. Arif, Muhammad, Saeed Ahmed, Fang Ge, Muhammad Kabir, Yaser Daanial Khan, Dong-Jun Yu, Maha Thafar. 2022. “StackACPred: Prediction of anticancer peptides by integrating optimized multiple feature descriptors with stacked ensemble approach.” *Chemometrics and Intelligent Laboratory Systems* 220: 104458. <https://doi.org/10.1016/j.chemolab.2021.104458>

28. Aziz, Abu Zahid Bin, Md. Al Mehedi Hasan, Shamim Ahmad, Md. Al Mamun, Jungpil Shin, Md Rahat Hossain. 2022. “iACP-MultiCNN: Multi-channel CNN based anticancer peptides identification.” *Analytical Biochemistry* 652: 114707. <https://doi.org/10.1016/j.ab.2022.114707>

29. Wu, Xiujin, Wenhua Zeng, Fan Lin. 2022. “GCNCPR-ACPs: a novel graph convolution network method for ACPs prediction.” *Bmc Bioinformatics* 23: 560. <https://doi.org/10.1186/s12859-022-04771-2>

30. Lv, Zhibin, Feifei Cui, Quan Zou, Lichao Zhang, Lei Xu. 2021. “Anticancer peptides prediction with deep representation learning features.” *Briefings in Bioinformatics* 22: bbab008. <https://doi.org/10.1093/bib/bbab008>

31. Wan, Yu, Zhuo Wang, Tzong-Yi Lee. 2021. “Incorporating support vector machine with sequential minimal optimization to identify anticancer peptides.” *Bmc Bioinformatics* 22: 286. <https://doi.org/10.1186/s12859-021-03965-4>

32. Agrawal, Piyush, Dhruv Bhagat, Manish Mahalwal, Neelam Sharma, Gajendra P. S. Raghava. 2021. “AntiCP 2.0: an updated model for predicting anticancer peptides.” *Briefings in Bioinformatics* 22: bbaa153. <https://doi.org/10.1093/bib/bbaa153>

33. Timmons, Patrick Brendan, Chandralal M. Hewage. 2021. “ENNAACT is a novel tool which employs neural networks for anticancer activity classification for therapeutic peptides.” *Biomedicine & Pharmacotherapy* 133: 111051. <https://doi.org/10.1016/j.biopha.2020.111051>

34. Chen, Xian-gan, Wen Zhang, Xiaofei Yang, Chenhong Li, Hengling Chen. 2021. “ACP-DA: Improving the prediction of anticancer peptides using data augmentation.” *Frontiers in Genetics* 12: 698477. <https://doi.org/10.3389/fgene.2021.698477>

35. Cai, Lijun, Li Wang, Xiangzheng Fu, Xiangxiang Zeng. 2021. “Active semisupervised model for improving the identification of anticancer peptides.” *Acs Omega* 6: 23998-24008. <https://doi.org/10.1021/acsomega.1c03132>

36. Nasiri, Farid, Fereshteh Fallah Atanaki, Saman Behrouzi, Kaveh Kavousi, Mojtaba Bagheri. 2021. “CpACpP: *In silico* cell-penetrating anticancer peptide prediction using a novel bioinformatics framework.” *Acs Omega* 6: 19846-19859. <https://doi.org/10.1021/acsomega.1c02569>

37. He, Wenjia, Yu Wang, Lizhen Cui, Ran Su, Leyi Wei. 2021. “Learning embedding features based on multisense-scaled attention architecture to improve the predictive performance of anticancer peptides.” *Bioinformatics* 37: 4684-4693. <https://doi.org/10.1093/bioinformatics/btab560>

38. Huang, Kai-Yao, Yi-Jhan Tseng, Hui-Ju Kao, Chia-Hung Chen, Hsiao-Hsiang Yang, Shun-Long Weng. 2021. “Identification of subtypes of anticancer peptides based on sequential features and physicochemical properties.” *Scientific Reports* 11: 13594. <https://doi.org/10.1038/s41598-021-93124-9>

39. Charoenkwan, Phasit, Wararat Chiangjong, Vannajan Sanghiran Lee, Chanin Nantasenamat, Md Mehedi Hasan, Watshara Shoombuatong. 2021. “Improved prediction and characterization of anticancer activities of peptides using a novel flexible scoring card method.” *Scientific Reports* 11: 3017. <https://doi.org/10.1038/s41598-021-82513-9>

40. Li, Qingwen, Wenyang Zhou, Donghua Wang, Sui Wang, Qingyuan Li. 2020. “Prediction of anticancer peptides using a low-dimensional feature model.” *Frontiers in Bioengineering and Biotechnology* 8: 892. <https://doi.org/10.3389/fbioe.2020.00892>

41. Ge, Ruiquan, Guanwen Feng, Xiaoyang Jing, Renfeng Zhang, Pu Wang, Qing Wu. 2020. “EnACP: An ensemble learning model for identification of anticancer peptides.” *Frontiers in Genetics* 11: 760. <https://doi.org/10.3389/fgene.2020.00760>

42. Rao, Bing, Chen Zhou, Guoying Zhang, Ran Su, Leyi Wei. 2020. “ACPred-Fuse: fusing multi-view information improves the prediction of anticancer peptides.” *Briefings in Bioinformatics* 21: 1846-1855. <https://doi.org/10.1093/bib/bbz088>

43. Zhao, Tianyi, Yang Hu, Tianyi Zang. 2020. “DRACP: a novel method for identification of anticancer peptides.” *Bmc Bioinformatics* 21: 559. <https://doi.org/10.1186/s12859-020-03812-y>

44. Rao, Bing, Lichao Zhang, Guoying Zhang. 2020. “ACP-GCN: The identification of anticancer peptides based on graph convolution networks.” *Ieee Access* 8: 176005-176011. <https://doi.org/10.1109/access.2020.3023800>

45. Akbar, Shahid, Ateeq Ur Rahman, Maqsood Hayat, Mohammad Sohail. 2020. “cACP: Classifying anticancer peptides using discriminative intelligent model via Chou's 5-step rules and general pseudo components.” *Chemometrics and Intelligent Laboratory Systems* 196: 103912. <https://doi.org/10.1016/j.chemolab.2019.103912>

46. Akbar, Shahid, Maqsood Hayat, Muhammad Tahir, Kil To Chong. 2020. “cACP-2LFS: Classification of anticancer peptides using sequential discriminative model of KSAAP and two-level feature selection approach.” *Ieee Access* 8: 131939-131948. <https://doi.org/10.1109/access.2020.3009125>

47. Schaduangrat, Nalini, Chanin Nantasenamat, Virapong Prachayasittikul, Watshara Shoombuatong. 2019. “ACPred: A computational tool for the prediction and analysis of anticancer peptides.” *Molecules* 24: 1973. <https://doi.org/10.3390/molecules24101973>

48. Boopathi, Vinothini, Sathiyamoorthy Subramaniyam, Adeel Malik, Gwang Lee, Balachandran Manavalan, Deok-Chun Yang. 2019. “mACPpred: A support vector machine-based meta-predictor for identification of anticancer peptides.” *International Journal of Molecular Sciences* 20: 1964. <https://doi.org/10.3390/ijms20081964>

49. Yue, Jianda, Tingting Li, Jiawei Xu, Zihui Chen, Yaqi Li, Songping Liang, Zhonghua Liu, Ying Wang. 2025. “Discovery of anticancer peptides from natural and generated sequences using deep learning.” *International Journal of Biological Macromolecules* 290: 138880. <https://doi.org/10.1016/j.ijbiomac.2024.138880>

50. Shahid, Maqsood, Maqsood Hayat, Wajdi Alghamdi, Shahid Akbar, Ali Raza, Rabiah Abdul Kadir, Mahidur R. Sarker. 2025. “pACP-HybDeep: predicting anticancer peptides using binary tree growth based transformer and structural feature encoding with deep-hybrid learning.” *Scientific Reports* 15: 565. <https://doi.org/10.1038/s41598-024-84146-0>

51. Lee, Byungjo, Dongkwan Shin. 2024. “Contrastive learning for enhancing feature extraction in anticancer peptides.” *Briefings in Bioinformatics* 25: bbae220. <https://doi.org/10.1093/bib/bbae220>

52. Xu, Xiaofang, Chaoran Li, Xinpu Yuan, Qiangjian Zhang, Yi Liu, Yunping Zhu, Tao Chen. 2024. “ACP-DRL: an anticancer peptides recognition method based on deep representation learning.” *Frontiers in Genetics* 15: 1376486. <https://doi.org/10.3389/fgene.2024.1376486>

53. Liu, Mingyou, Tao Wu, Xue Li, Yingxue Zhu, Sen Chen, Jian Huang, Fengfeng Zhou, Hongmei Liu. 2024. “ACPPfel: Explainable deep ensemble learning for anticancer peptides prediction based on feature optimization.” *Frontiers in Genetics* 15: 1352504. <https://doi.org/10.3389/fgene.2024.1352504>

54. Karakaya, Onur, Zeynep Hilal Kilimci. 2024. “An efficient consolidation of word embedding and deep learning techniques for classifying anticancer peptides: FastText+BiLSTM.” *Peerj Computer Science* 10: e1831. <https://doi.org/10.7717/peerj-cs.1831>

55. Khan, Shujaat. 2024. “Deep-representation-learning-based classification strategy for anticancer peptides.” *Mathematics* 12: 1330. <https://doi.org/10.3390/math12091330>

56. Zhang, Ming, Jianren Zhou, Xiaohua Wang, Xun Wang, Fang Ge. 2024. “DeepBP: Ensemble deep learning strategy for bioactive peptide prediction.” *Bmc Bioinformatics* 25: 352. <https://doi.org/10.1186/s12859-024-05974-5>

57. Chen, Nanjun, Jixiang Yu, Liu Zhe, Fuzhou Wang, Xiangtao Li, Ka-Chun Wong. 2024. “TP-LMMSG: a peptide prediction graph neural network incorporating flexible amino acid property representation.” *Briefings in Bioinformatics* 25: bbae308. <https://doi.org/10.1093/bib/bbae308>

58. Wang, Xin, Zimeng Zhang, Chang Liu. 2024. “iACP-DFSRA: Identification of anticancer peptides based on a dual-channel fusion strategy of resCNN and attention.” *Journal of Molecular Biology* 436: 168810. <https://doi.org/10.1016/j.jmb.2024.168810>

59. Tao, Huawei, Shuai Shan, Hongliang Fu, Chunhua Zhu, Boye Liu. 2023. “An augmented sample selection framework for prediction of anticancer peptides.” *Molecules* 28: 6680. <https://doi.org/10.3390/molecules28186680>

60. Sun, Mingwei, Sen Yang, Xuemei Hu, You Zhou. 2022. “ACPNet: A deep learning network to identify anticancer peptides by hybrid sequence information.” *Molecules* 27: 1544. <https://doi.org/10.3390/molecules27051544>

61. Sun, Yih-Yun, Tzu-Tang Lin, Wen-Chih Cheng, I-Hsuan Lu, Chung-Yen Lin, Shu-Hwa Chen. 2022. “Peptide-based drug predictions for cancer therapy using deep learning.” *Pharmaceuticals* 15: 422. <https://doi.org/10.3390/ph15040422>

62. Ghulam, Ali, Farman Ali, Rahu Sikander, Ashfaq Ahmad, Aftab Ahmed, Shruti Patil. 2022. “ACP-2DCNN: Deep learning-based model for improving prediction of anticancer peptides using two-dimensional convolutional neural network.” *Chemometrics and Intelligent Laboratory Systems* 226: 104589. <https://doi.org/10.1016/j.chemolab.2022.104589>

63. Han, Bingqing, Nan Zhao, Chengshi Zeng, Zengchao Mu, Xinqi Gong. 2022. “ACPred-BMF: bidirectional LSTM with multiple feature representations for explainable anticancer peptide prediction.” *Scientific Reports* 12: 21915. <https://doi.org/10.1038/s41598-022-24404-1>

64. Cao, Ruifen, Meng Wang, Yannan Bin, Chunhou Zheng. 2021. “DLFF-ACP: prediction of ACPs based on deep learning and multi-view features fusion.” *Peerj* 9: e11906. <https://doi.org/10.7717/peerj.11906>

65. Chen, Jiarui, Hong Hin Cheong, Shirley W., I Siu. 2021. “xDeep-AcPEP: Deep learning method for anticancer peptide activity prediction based on convolutional neural network and multitask learning.” *Journal of Chemical Information and Modeling* 61: 3789-3803. <https://doi.org/10.1021/acs.jcim.1c00181>

66. Yu, Lezheng, Runyu Jing, Fengjuan Liu, Jiesi Luo, Yizhou Li. 2020. “DeepACP: A novel computational approach for accurate identification of anticancer peptides by deep learning algorithm.” *Molecular Therapy-Nucleic Acids* 22: 862-870. <https://doi.org/10.1016/j.omtn.2020.10.005>

67. Yi, Hai-Cheng, Zhu-Hong You, Xi Zhou, Li Cheng, Xiao Li, Tong-Hai Jiang, Zhan-Heng Chen. 2019. “ACP-DL: A deep learning long short-term memory model to predict anticancer peptides using high-efficiency feature representation.” *Molecular Therapy Nucleic Acids* 17: 1-9. <https://doi.org/10.1016/j.omtn.2019.04.025>

68. Kabir, Muhammad, Muhammad Arif, Saeed Ahmad, Zakir Ali, Zar Nawab Khan Swati, Dong-Jun Yu. 2018. “Intelligent computational method for discrimination of anticancer peptides by incorporating sequential and evolutionary profiles information.” *Chemometrics and Intelligent Laboratory Systems* 182: 158-165. <https://doi.org/10.1016/j.chemolab.2018.09.007>

69. Tyagi, Atul, Abhishek Tuknait, Priya Anand, Sudheer Gupta, Minakshi Sharma, Deepika Mathur, Anshika Joshi, Sandeep Singh, Ankur Gautam, Gajendra P. S. Raghava. 2015. “CancerPPD: a database of anticancer peptides and proteins.” *Nucleic Acids Research* 43: D837-D843. <https://doi.org/10.1093/nar/gku892>

70. Pirtskhalava, Malak, Anthony A. Amstrong, Maia Grigolava, Mindia Chubinidze, Evgenia Alimbarashvili, Boris Vishnepolsky, Andrei Gabrielian, Alex Rosenthal, Darrell E. Hurt, Michael Tartakovsky. 2021. “DBAASP v3: database of antimicrobial/cytotoxic activity and structure of peptides as a resource for development of new therapeutics.” *Nucleic Acids Research* 49: D288-D297. <https://doi.org/10.1093/nar/gkaa991>

71. Wang, Guangshun, Xia Li, Zhe Wang. 2016. “APD3: the antimicrobial peptide database as a tool for research and education.” *Nucleic Acids Research* 44: D1087-D1093. <https://doi.org/10.1093/nar/gkv1278>

72. Gawde, Ulka, Shuvechha Chakraborty, Faiza Hanif Waghu, Ram Shankar Barai, Ashlesha Khanderkar, Rishikesh Indraguru, Tanmay Shirsat, Susan Idicula-Thomas. 2023. “CAMP_R4_: a database of natural and synthetic antimicrobial peptides.” *Nucleic Acids Research* 51: D377-D383. <https://doi.org/10.1093/nar/gkac933>

73. Novkovic, Mario, Juraj Simunic, Viktor Bojovic, Alessandro Tossi, Davor Juretic. 2012. “DADP: the database of anuran defense peptides.” *Bioinformatics* 28: 1406-1407. <https://doi.org/10.1093/bioinformatics/bts141>

74. Yao, Lantian, Jiahui Guan, Peilin Xie, Chia-Ru Chung, Zhihao Zhao, Danhong Dong, Yilin Guo, et al. 2024. “dbAMP 3.0: updated resource of antimicrobial activity and structural annotation of peptides in the post-pandemic era.” *Nucleic Acids Research* 53: D364-D376. <https://doi.org/10.1093/nar/gkae1019>

75. Ma, Tianyue, Yanchao Liu, Bingxin Yu, Xin Sun, Huiyuan Yao, Chen Hao, Jianhui Li, et al. 2024. “DRAMP 4.0: an open-access data repository dedicated to the clinical translation of antimicrobial peptides.” *Nucleic Acids Research* 53: D403-D410. <https://doi.org/10.1093/nar/gkae1046>

76. Singh, Sandeep, Kumardeep Chaudhary, Sandeep Kumar Dhanda, Sherry Bhalla, Salman Sadullah Usmani, Ankur Gautam, Abhishek Tuknait, Piyush Agrawal, Deepika Mathur, Gajendra P. S. Raghava. 2016. “SATPdb: a database of structurally annotated therapeutic peptides.” *Nucleic Acids Research* 44: D1119-D1126. <https://doi.org/10.1093/nar/gkv1114>

77. Li, Qilin, Chao Zhang, Hongjun Chen, Jitong Xue, Xiaolei Guo, Ming Liang, Ming Chen. 2018. “BioPepDB: an integrated data platform for food-derived bioactive peptides.” *International Journal of Food Sciences and Nutrition* 69: 963-968. <https://doi.org/10.1080/09637486.2018.1446916>

78. Das, Durdam, Mohini Jaiswal, Fatima Nazish Khan, Shahzaib Ahamad, Shailesh Kumar. 2020. “PlantPepDB: A manually curated plant peptide database.” *Scientific Reports* 10: 2194. <https://doi.org/10.1038/s41598-020-59165-2>

79. Zareei, Sara, Babak Khorsand, Alireza Dantism, Neda Zareei, Fereshteh Asgharzadeh, Shadi Shams Zahraee, Samane Mashreghi Kashan, et al. 2024. “PeptiHub: a curated repository of precisely annotated cancer-related peptides with advanced utilities for peptide exploration and discovery.” *Database-the Journal of Biological Databases and Curation* 2024: baae092. <https://doi.org/10.1093/database/baae092>

80. Wang, Fangyu, Ning Li, Chunfeng Wang, Guangxu Xing, Shuai Cao, Qian Xu, Yunshang Zhang, Man Hu, Gaiping Zhang. 2020. “DPL: a comprehensive database on sequences, structures, sources and functions of peptide ligands.” *Database-the Journal of Biological Databases and Curation* 2020: baaa089. <https://doi.org/10.1093/database/baaa089>

81. Cabas-Mora, Gabriel, Anamaria Daza, Nicole Soto-García, Valentina Garrido, Diego Alvarez, Marcelo Navarrete, Lindybeth Sarmiento-Varón, et al. 2024. “Peptipedia v2.0: a peptide sequence database and user-friendly web platform. A major update.” *Database-the Journal of Biological Databases and Curation* 2024: baae113. <https://doi.org/10.1093/database/baae113>

82. Faraji, Naser, Seyed Shahriar Arab, Alireza Doustmohammad, Norelle L. Daly, Ahmad Yari Khosroushahi. 2022. “ApInAPDB: a database of apoptosis-inducing anticancer peptides.” *Scientific Reports* 12: 21341. <https://doi.org/10.1038/s41598-022-25530-6>

83. Kapoor, Pallavi, Harinder Singh, Ankur Gautam, Kumardeep Chaudhary, Rahul Kumar, Gajendra P. S. Raghava. 2012. “TumorHoPe: A database of tumor homing peptides.” *PLoS ONE* 7: e35187. <https://doi.org/10.1371/journal.pone.0035187>

84. Jain, Shipra, Srijanee Gupta, Sumeet Patiyal, Gajendra P. S. Raghava. 2024. “THPdb2: compilation of FDA approved therapeutic peptides and proteins.” *Drug Discovery Today* 29: 104047. <https://doi.org/10.1016/j.drudis.2024.104047>

85. Nielsen, Soren Drud, Robert L. Beverly, Yunyao Qu, David C. Dallas. 2017. “Milk bioactive peptide database: A comprehensive database of milk protein-derived bioactive peptides and novel visualization.” *Food Chemistry* 232: 673-682. <https://doi.org/10.1016/j.foodchem.2017.04.056>

86. Minkiewicz, Piotr, Anna Iwaniak, Malgorzata Darewicz. 2019. “BIOPEP-UWM database of bioactive peptides: Current opportunities.” *International Journal of Molecular Sciences* 20: 5978. <https://doi.org/10.3390/ijms20235978>
